# Determinants of diabetic retinopathy in Ethiopia: A systematic review and meta-analysis

**DOI:** 10.1371/journal.pone.0286627

**Published:** 2023-06-08

**Authors:** Abere Woretaw Azagew, Yeneabat Birhanu Yohanes, Zerko Wako Beko, Yohannes Mulu Ferede, Chilot Kassa Mekonnen

**Affiliations:** 1 Department of Medical Nursing, School of Nursing, College of Medicine and Health Sciences, University of Gondar, Gondar, Ethiopia; 2 Department of Surgical Nursing, School of Nursing, College of Medicine and Health Sciences, University of Gondar, Gondar, Ethiopia; University of the Highlands and Islands, UNITED KINGDOM

## Abstract

**Introduction:**

Diabetic retinopathy (DR) is the primary retinal vascular complication of diabetes mellitus and a leading cause of visual impairment and blindness. It affects the global diabetic population. In Ethiopia, about one-fifth of diabetic patients were affected by DR, but there were inconsistent finding across studies about the determinants factors of DR. Therefore, we aimed to identify the risk factors for DR among diabetic patients.

**Methods:**

We have accessed previous studies through an electronic web-based search strategy using PubMed, Google (Scholar), the Web of Science, and the Cochrane Library with a combination of search terms. The quality of each included article was assessed using the Newcastle Ottawa Assessment Scale. All statistical analyses were carried out using Stata version 14 software. The odds ratios of risk factors were pooled using a fixed-effect meta-analysis model. Heterogeneity was assessed using the Cochrane Q statistics and I-Square (I^2^). Furthermore, publication bias was detected based on the graphic asymmetry test of the funnel plot and/or Egger’s test (p< 0.05).

**Results:**

The search strategy retrieved 1285 articles. After the removal of duplicate articles, 249 articles remained. Following further screening, about 18 articles were assessed for eligibility, of which three articles were excluded because of reporting without the outcome of interest, poor quality, and not full text. Finally, fifteen studies were reviewed for the final analysis. Co-morbid hypertension (HTN) (AOR 2.04, 95%CI: 1.07, 3.89), poor glycemic control (AOR = 4.36, 95%CI: 1.47, 12.90), and duration of diabetes illness (AOR = 3.83, 95%CI: 1.17, 12.55) were found to be confirmed associated factors of diabetic retinopathy.

**Conclusion:**

In this study, co-morbid HTN, poor glycemic control, and longer duration of diabetes illness were found to be the determinant factors of DR. Aggressive treatment of co-morbid HTN and blood glucose, and regular eye screening should be implemented to reduce the occurrence of DR.

**Trial registration:**

The review protocol was registered in the international prospective register of systematic reviews (PROSPERO) with registration number PROSPERO: CRD42023416724.

## Introduction

Diabetic retinopathy is a diabetic complication that affects the eyes. It is caused by damage to the blood vessels of the light-sensitive tissue at the back of the eyes [1]. Diabetic retinopathy is recognized as microvascular complication [2]. Clinically, diabetic retinopathy is classified as non-proliferative and proliferative diabetic retinopathy. Non-proliferative diabetic retinopathy (NPDR) is the earliest and the asymptomatic stage whereas proliferative diabetic retinopathy (PDR) is the advanced stage of diabetic retinopathy characterized by neovascularization. In the PDM stage, the patient experiences severe vision impairment when the new abnormal blood vessel bleeds to the vitreous (vitreous hemorrhage) or retinal detachment [3]. The most common cause of vision loss in patients with diabetic retinopathy is diabetic macular edema. It is characterized by swelling or thickening of the macula which causes distortion of visual images and a decrease in visual acuity [4, 5].

Diabetic retinopathy is the leading cause of visual impairment or blindness among the working-age population in the world. It is considered the third leading cause of blindness worldwide [6]. It affects both type 1& 2 diabetic patients, but its incidence is higher in type 1 than in type 2 diabetes mellitus populations [7].

The global prevalence of DR was (22.27%) [8], Africa (33.8%) [6], and Ethiopia (19.48%) [9]. The pathogenesis of diabetic retinopathy is not well known, but it is associated with high blood glucose. Overtime, having too much sugar in the blood can damage the retina vasculature. In the earlier stage, it forms microaneurysm, capillary leakage, retinal edema, capillary occlusion, ischemia, and cotton wool spot formation. If it is not treated, it results in loss of vision [10, 11].

Even though prevention strategies such as keeping blood glucose to the optimum level, adopting a healthy lifestyle, losing weight, exercising regularly, and health education to adhere to medications [12, 13] implemented in diabetic follow-up care to reduce the incidence of diabetic retinopathy, but the problem is still rising [14]. Therefore, risk stratification and selective early intervention for high-risk patients need to be given attention. In Ethiopia, there were different research articles reporting on the determinant factors of diabetic retinopathy, but their findings were inconsistent across the studies. Therefore, this study aimed to identify the determinant factors of DR among diabetic patients.

## Methods

### Reporting

The review protocol has been registered in the international prospective register of systematic reviews (PROSPERO) with registration number (PROSPERO: CRD42023416724), and the result of the review was presented based on standard Preferred Reporting Items for Systematic review and Meta-analysis (PRISMA) [15] checklist (**S1 File**).

### Study selection and search strategy

The procedure for this systematic review and meta-analysis was designed following the Preferred Reporting Items for Systematic Review and Meta-Analysis (PRISMA) flow chart [15]. We searched on PubMed, Google (for grey literature), Google Scholar, Web of Science, and Cochrane Library databases for studies reporting diabetic retinopathy. Endnote (Version 7) reference management software was used to download, organize, review, de-duplicate, and cite the articles. Our comprehensive search strategies were carried out using controlled vocabularies (MeSH terms). Using the MeSH database, the synonyms of diabetic retinopathy were identified. Then, the search string was established using the databases. Articles were searched by title (Ti), abstract (Ab), full text, or all these categories. Modification of the search strategy was made by limiters such as study design, and country. Boolean logic operators such as “AND” and “OR” were used to combine searching terms. The search strings were stated as: "diabetic retinopathy" OR "diabetic retinopathy*" OR "diabetic eye complication" OR "diabetic macular edema" OR diabetic macular? edema OR "diabetic angiopathy" OR "diabetic angiopath?" AND “adult diabetic patients” OR “diabetes mellitus patients” AND Ethiopia. Two reviewers independently searched and screened articles by title, abstract, and full text. The disagreements between the reviewers were resolved by discussion.

### Inclusion and exclusion criteria

The eligibility of the included studies was summarized in the table below (**Table 1**).

**Table 1:** Inclusion and exclusion criteria for included research articles.

Criteria	Inclusion criteria	Exclusion criteria
Participants	People with diabetic retinopathy age ≥18 years	Population with no outcome interest
Study setting	Hospital or health facility	Community-based study
Design	Observational study designs (cross-sectional, cohort, and case-control)	
Publication status	both published and unpublished studies	Qualitative studies conference papers articles with no full text
Language of publication	English	Languages other than the English language
Country	Ethiopia different regions of the country	
Publication year	No restriction	

### Data extraction

The data were extracted by data abstraction format using the Microsoft excel spreadsheet. The format was developed by two reviewers and piloted for its clarity, aim, consistency, and depth of the contents. Simple and consistent codes of response were used. Then the reviewers independently reviewed and extract data from each eligible study. The information such as authors, publication year, region of the study, design, methodological quality, population, study setting, sample size, method of data collection, statistical analysis, and funding source were extracted from the studies (**S2 File**).

### Quality assessment

Articles were assessed for quality score using the New Castle Ottawa Scale adapted from cross-sectional, cohort, and case control’ quality assessment tools; a score of ≥ 7 out of 10 was considered a high-quality score [16]. Two reviewers (CKM and HMA) assessed the quality of each paper. The reviewers compared the quality of the appraisal scores and resolved inconsistencies before calculating the final appraisal score. All the included studies had the high-quality scores. The PRISMA checklist 2020 [17] was used to report the results of this study.

### Data analysis

The data were entered to a Microsoft excel spreadsheet and exported to Stata version 14 for analysis. Cochran’s Q statistic and I-squared (I^2^) were used to evaluate the presence of heterogeneity. The I-square test statistic results of 25%, 50%, and 75% were declared as low, moderate, and high heterogeneity [18], respectively. The pooled summary effect size was estimated using the fixed effect model [19]. The publication bias was detected based on the graphic asymmetry test of the funnel plot and/or Egger’s test (p< 0.05) [20].

## Results

### Study selection and characteristics

**Fig 1:**
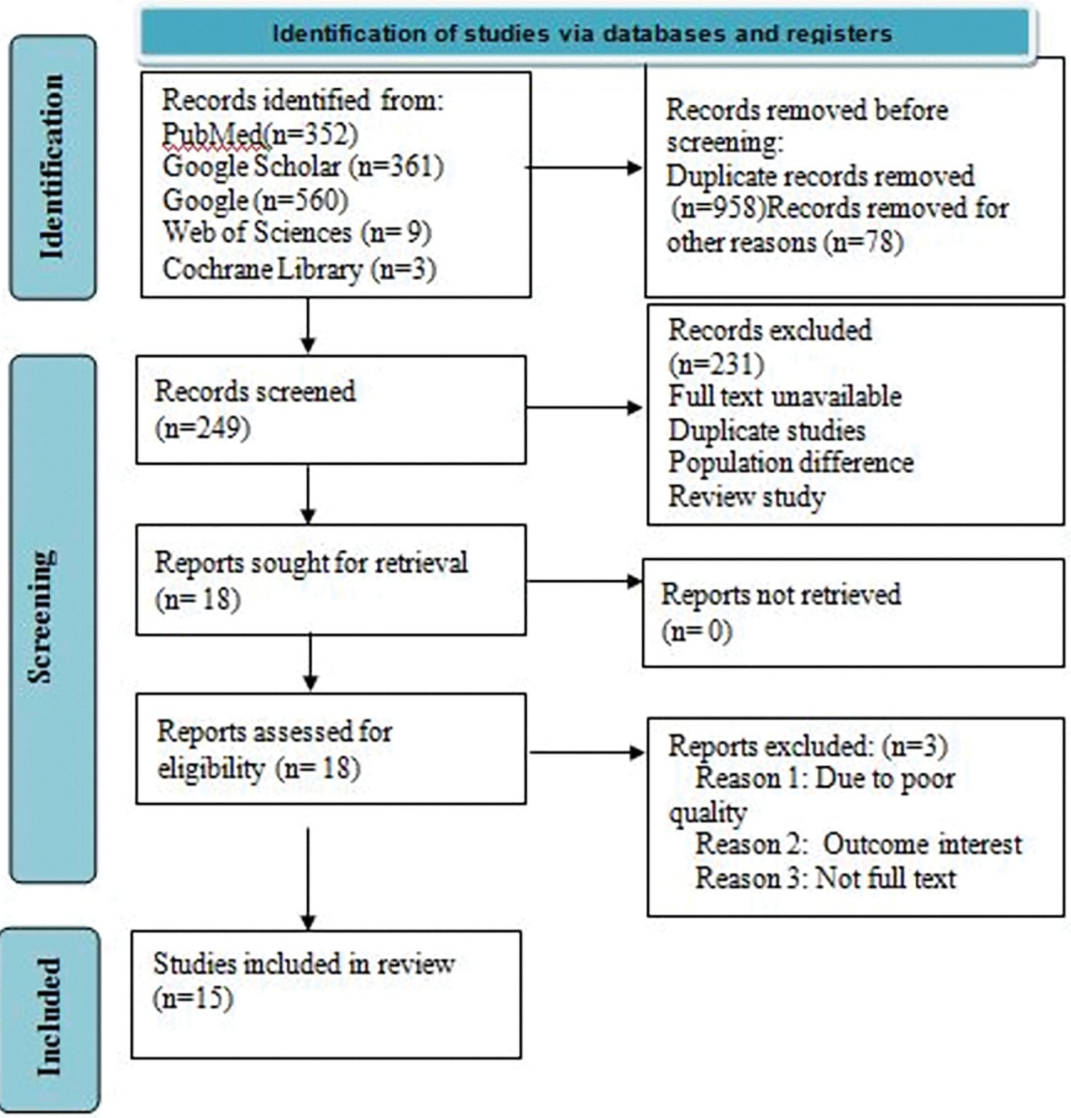
PRISMA flow chart for flow of information through the phase of systematic review.

The search strategy retrieved 1285 articles. After the removal of duplicate articles, 249 articles remained. Following the additional screening, eighteen articles were evaluated for eligibility, with three being excluded due to being incomplete, of poor quality, or not in full text (**Fig 1**). Finally, fifteen studies were reviewed for co-morbid HTN [21–35] (**Table 2**), six studies for poor glycemic control [21, 22, 24, 25, 28, 32] (**Table 3**), and five studies for the duration of diabetic illness [21, 22, 25, 27, 31] (**Table 4**). Of the total studies; five were conducted in the Amhara region [21, 23, 27, 30, 34], five in Addis Ababa [24, 26, 29, 32, 35], three in the Oromia region [25, 31, 33], and two in South Nations and Nationalities people (SNNP) of Ethiopia [22, 28].

**Table 2:** Study characteristics by co-morbid HTN among diabetic patients in Ethiopia.

Author/year	Study design	Region of the study	data collection technique	Funding Source	Factors	AOR	95% CI	overall quality score
Tilahun M, et al./2020 [21]	Cross-sectional	Amhara	Interview & patient review	not funded	Co-HTN	3.39	1.64–7.02	8.5
Alemayehu HB, et al./2022 [22]	Cross-sectional	SNNP	Interview& patient chart review	not reported	Co-HTN	1.43	0.72–2.86	7.4
Mersha GA, et al./2021 [23]	Cross-sectional	Amhara	interview & chart review	not reported	Co-HTN	1.67	0.66–4.20	7
Seid K, et al./2021 [24]	case-control	Addis Ababa	interview & chart review	Jimma University, Institute of Health	Co-HTN	12.3	6.95–21.8	7.5
Garoma D, et al./2020 [25]	Case-control	Oromia	interview & chart review Ocular exam	Jimma University, Institute of Health	Co-HTN	3.38	1.29–9.05	7.0
Shiberu T, et al./2018 [26]	Cross-sectional	Addis Ababa	interview & chart review Ocular exam	Not reported	Co-HTN	2.556	1.014–6.447	9.0
Ejigu T, et al./2021 [27]	Cross-sectional	Amhara	interview & chart review Ocular exam	Not reported	Co-HTN	2.65	1.02–6.87	8.0
Chisha Y, et al./2017 [28]	Cohort	SNNP	Record review	Mekelle University	Co-HTN	4.1	1.76–9.44	7.5
Azeze TK, et al./2018 [29]	Cohort	Addis Ababa	Record review	self sponsored	Co-HTN	1.51	0.48–4.74	8.5
Takele MB, et al./2022 [30]	Cohort	Amhara	Record review	Amhara regional state	Co-HTN	1.68	1.14–2.50	8.4
Gelcho GN, et al./2022 [31]	Cohort	Oromia	Record review	Not Funded	Co-HTN	2.32	1.12–4.39	7.6
Aberra T, et al./2022 [32]	Cross-sectional	Addis Ababa	Interview Record review	Not reported	Co-HTN	1.37	0.865–2.169	8.5
Debele GR, et al./2021 [33]	Cohort	Oromia	Record review	University of Gondar	Co-HTN	0.54	0.35–0.82	8.0
Alemu S, et al./2015 [34]	Cross-sectional	Amhara	Interview & record review	not reported	Co-HTN	5.2	2.5–10.20	7.5
Abera F, et al./2021 [35]	Cross-sectional	Addis Ababa	interview & chart review ocular exam	Not reported	Co-HTN	8.63	2.51–29.75	7.0

**Notes**; AOR; Adjusted odds ratio, CI; Confidence Interval, Co-HTN: Co-morbid Hypertension, SNNP: Southern Nations, Nationalities and Peoples’

**Table 3:** Study characteristics by poor glycemic control status among diabetic patients in Ethiopia.

Author/year	Study design	Region of the study	Factors	AOR	95% CI
Tilahun M, et al./2020 [21]	Cross-sectional	Amhara	PGC	4.58	1.86–11.31
Alemayehu HB, et al./2022 [22]	Cross-sectional	SNNP	PGC	4.34	2.26–8.34
Mersha GA, et al./2021 [23]	Cross-sectional	Amhara	PGC	3.2	1.5–6.7
Seid K, et al./2021 [24]	case-control	Addis Ababa	PGC	10.7	6.17–18.58
Garoma D et al./2020 [25]	Case-control	Oromia	PGC	9.08	3.7–22.29
Aberra T, et al./2022 [32]	Cross-sectional	Addis Ababa	PGC	1.23	0.667–2.276

**Notes;** AOR; Adjusted odds ratio, CI; Confidence Interval, PGC: Poor glycemic control, SNNP: Southern Nations, Nationalities and Peoples’

**Table 4:** Study characteristics by duration of diabetic illness among diabetic patients in Ethiopia.

Author/year	Study design	Region of the study	Factors	AOR	95% CI
Tilahun M, et al./2020 [21]	Cross-sectional	Amhara	Duration Diabetic illness	3.91	1.86–8.23
Alemayehu HB, et al./2022 [22]	Cross-sectional	SNNP	Duration Diabetic illness	4.78	2.11–10.83
Garoma D, et al./2020 [25]	Case-control	Oromia	Duration Diabetic illness	4.38	2.65–7.22
Ejigu T, et al./2021 [27]	Cross-sectional	Amhara	Duration Diabetic illness	2.91	1.01–8.35
Gelcho GN, et al./2022 [31]	Cohort	Oromia	Duration Diabetic illness	2.86	1.41–5.31

**Notes;** AOR; Adjusted odds ratio, CI; Confidence Interval, PGC- Poor glycemic control, SNNP: Southern Nations, Nationalities and Peoples’

All the studies were published between the years 2015 and 2022. Regarding the study design, eight studies were cross-sectional [21–23, 26, 27, 32, 34, 35], five studies were cohort [28–31, 33], and two studies were case-control [24, 25]. The overall quality score of the included studies’ was ≥7.

## Determinants factors of diabetic retinopathy

### Co-morbid hypertension

**Fig 2:**
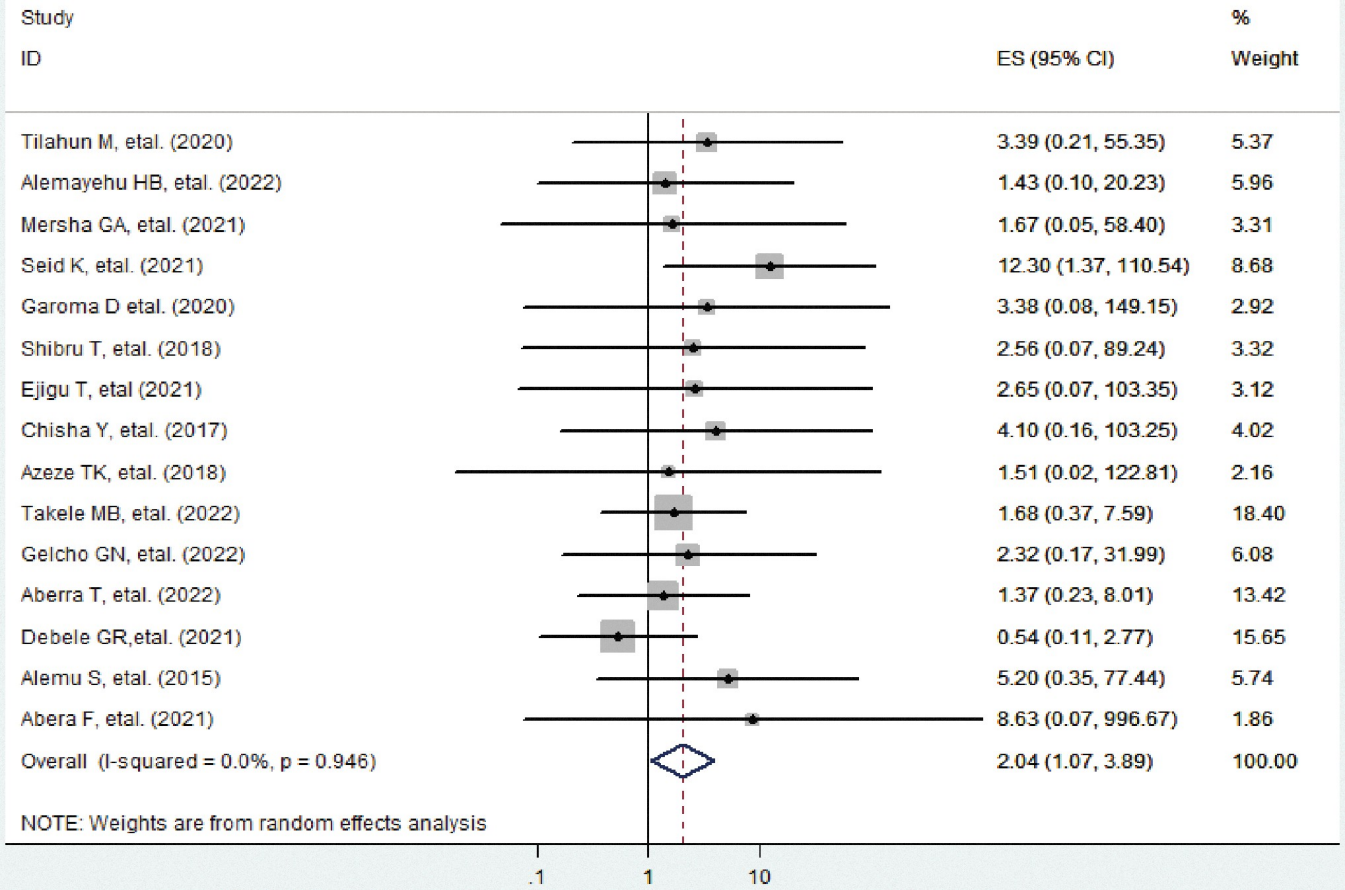
Forest plot shows the pooled effect of co-morbid hypertension on diabetic retinopathy.

In this systematic review and meta-analysis, co-morbid HTN is found to be the determinant factor for diabetic retinopathy. Diabetic patients who have co-morbid HTN are 2.04 times more likely to have diabetic retinopathy compared to those diabetic patients with no co-morbid HTN (AOR 2.04, 95%CI: 1.07, 3.89) (**Fig 2**).

### Heterogeneity and publication bias of included studies

The overall heterogeneity test (**I**^**2**^) on the effect of co-morbid HTN was 0.0% with a p-value < 0.946, using a random effect model to adjust observed variability. This indicates there is no variability across the studies.

**Fig 3:**
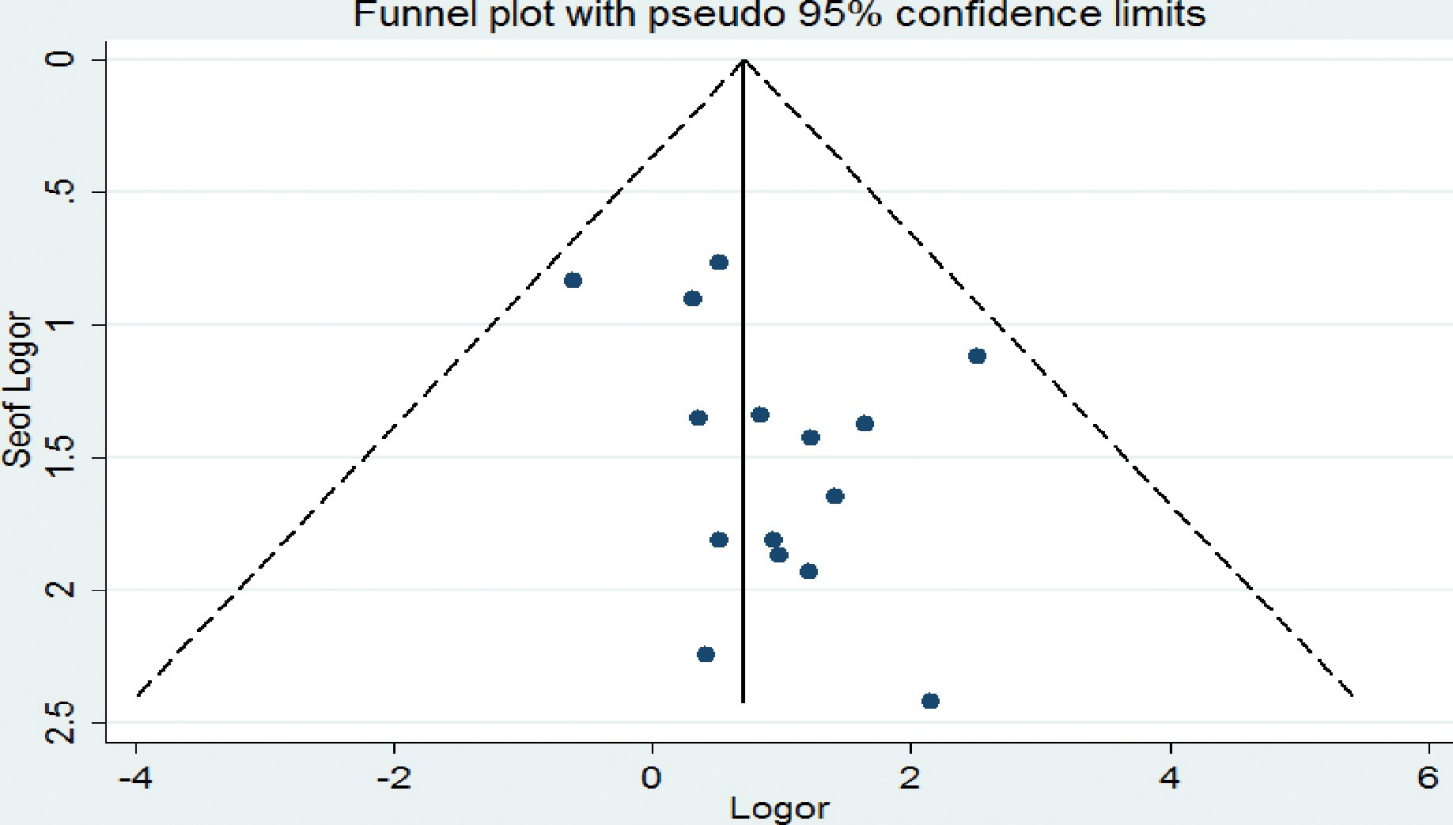
Funnel plot to assess the heterogeneity of the included studies.

Regarding the publication bias, the graphic asymmetry test of the funnel plot which shows a symmetrical distribution (**Fig 3**), and Egger’s test p-value = 0.181, indicating that there is no publication bias.

### Poor glycemic control

**Fig 4:**
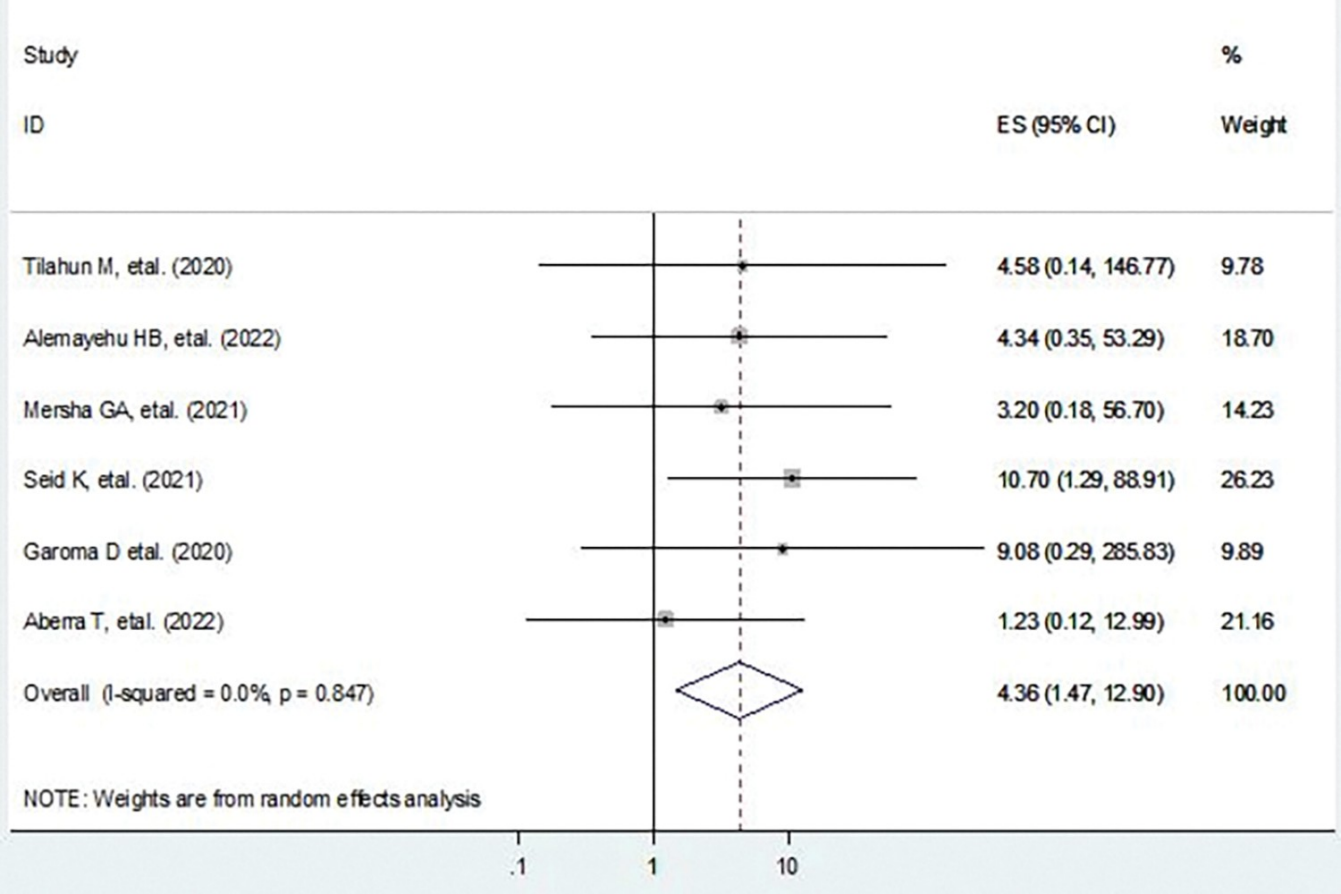
Forest plot shows the pooled effect of poor glycemic control on diabetic retinopathy.

In this study, poor glycemic control was found to be the determinant factor for diabetic retinopathy. Diabetic patients with poor glycemic control status were 4.36 times more likely to have diabetic retinopathy compared to those diabetic patients with good glycemic control status (AOR = 4.36, 95%CI: 1.47, 12.90) (**Fig 4**).

### Heterogeneity and publication bias of included studies

**Fig 5:**
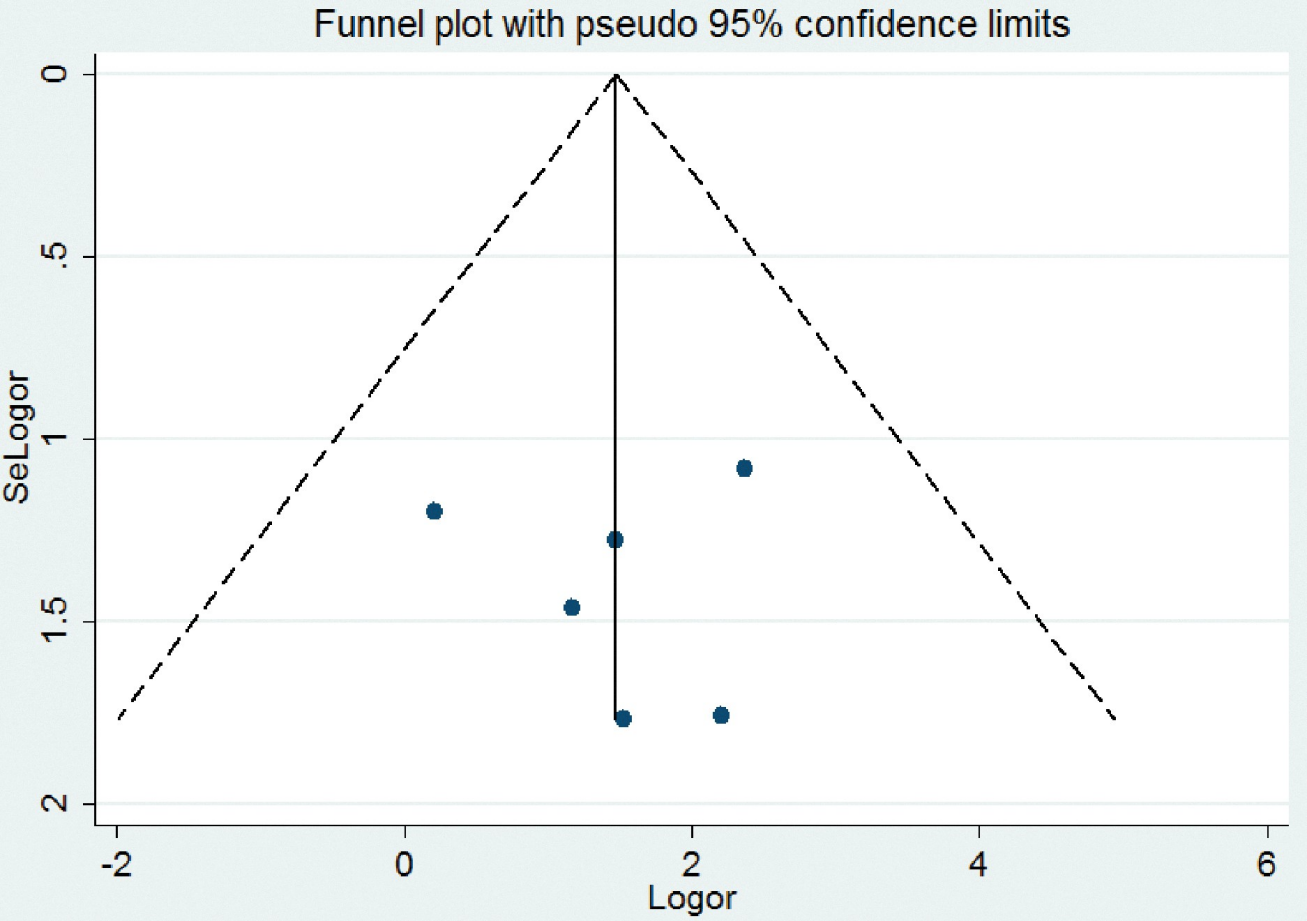
Funnel plot to assess the heterogeneity of the included studies.

The heterogeneity test (**I**^**2**^) on the effect of poor glycemic control was 0.0% with a p value< 0.847, using a random effect model to adjust observed variability. This indicates there is no variability across the studies. The publication bias was assessed using the graphic asymmetry test of the funnel plot, shows a symmetrical distribution (**Fig 5**), and Egger’s test p value = 0.851means that there was no publication bias.

### Duration of diabetic illness

**Fig 6:**
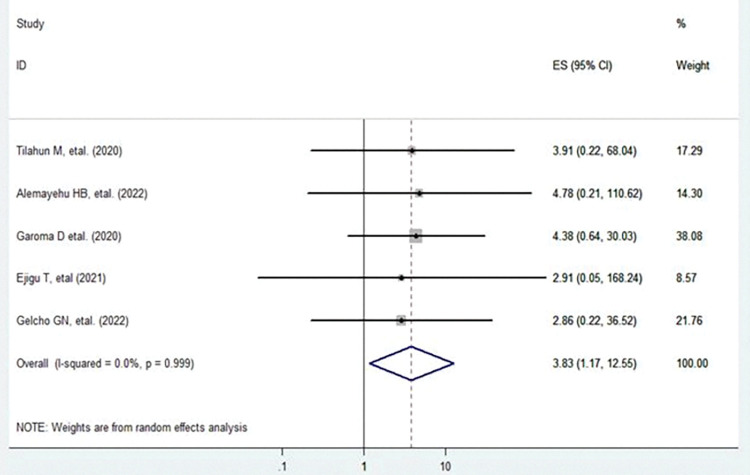
Forest plot shows the pooled effect of duration of diabetic illness on diabetic retinopathy.

Longer duration of diabetic illness is found to be the risk factor for the development of diabetic retinopathy. Patients who had a longer duration of diabetic illness were nearly four times more likely to have diabetic retinopathy compared to those diabetic patients with a shorter duration of diabetic illness (AOR = 3.83, 95%CI: 1.17, 12.55, I^2^ = 0.0% and p-value = 0.999) (**Fig 6**).

### Heterogeneity and publication bias of included studies

**Fig 7:**
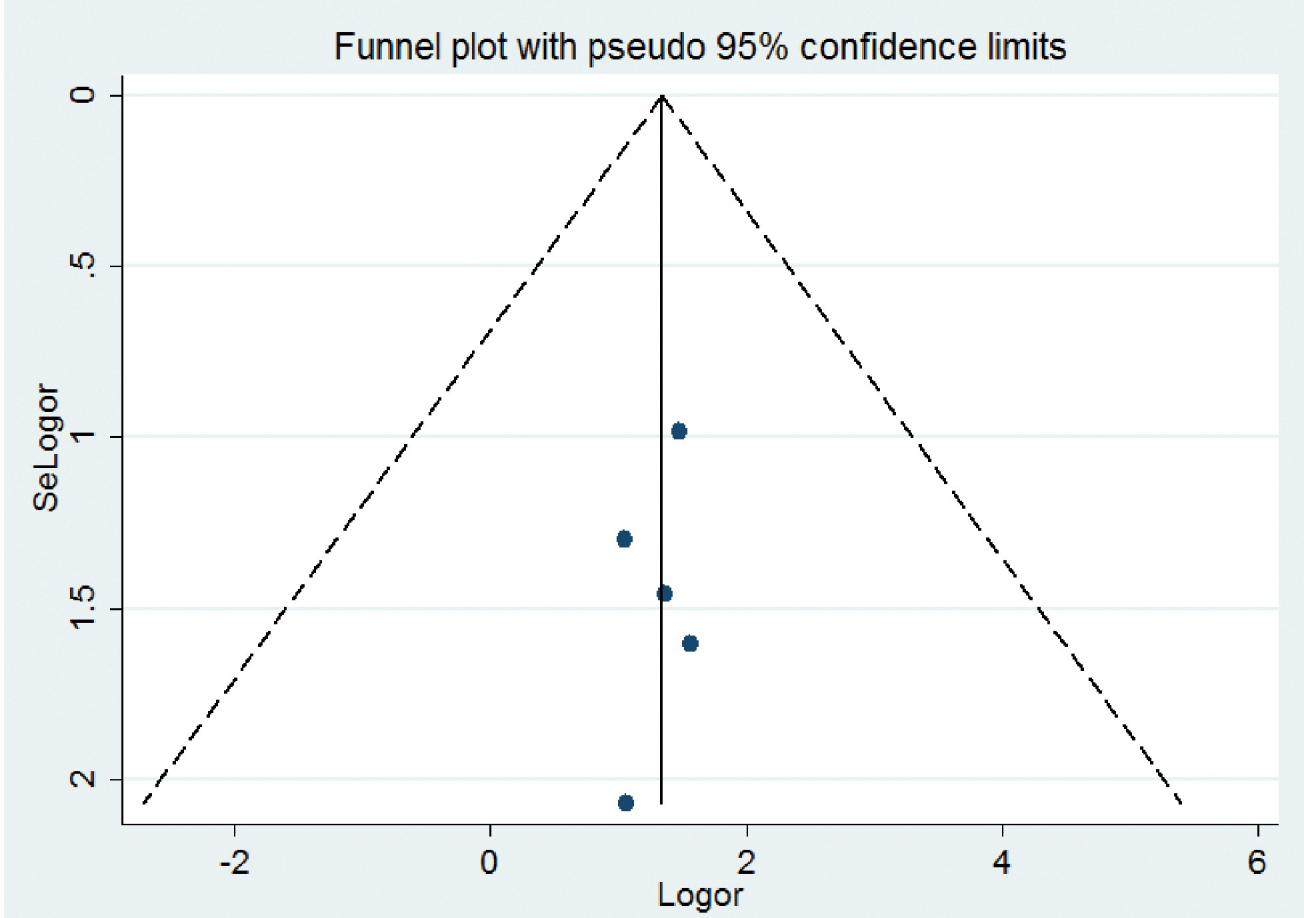
Funnel plot to assess the heterogeneity of the included studies.

The overall heterogeneity test (**I**^**2**^) on the effect of co-morbid hypertension was 0.0% with a p-value< 0.999, using a random effect model to adjust observed variability. This indicates there was no variability across the studies. Concerning the publication bias, the graphic asymmetry test of the funnel plot shows a symmetrical distribution (**Fig 7**), and Egger’s test p-value = 0.624, indicating that there is no publication bias.

## Discussion

Diabetic patients experience different complications along the continuum of their lives, of which diabetic retinopathy is the most common. We attempted to investigate the determinant factors of diabetic retinopathy. In this systematic review and meta-analysis, co-morbid HTN, poor glycemic control status, and longer duration of diabetic illness were found to be the determinate factors of diabetic retinopathy.

The pooled effect of this study depicts that diabetic patients who have co-morbid HTN were 2.04 times more likely to have diabetic retinopathy compared to those diabetic patients with no co-morbid HTN. The finding of this study is supported by large-scale studies in China [36], and (Chinese, Malay, and India) [37]. This is the fact that HTN has a direct impact on retinal blood vessels. It damages the retinal vascular structures [38]. The elevated blood pressure is transferred directly to the vessels, which initially constrict, but a further increase in BP overcomes this compensatory tone, and damage to the muscle layer and endothelium ensues [39]. This results in retinal edema, cottonwool spots, hemorrhage, and disc edema [40]. Evidence showed that tight blood pressure control in the diabetic population reduces the incidence of sight-threatening retinopathy with a favorable impact on the lives of diabetic patients. A decrease in every 10 mmHg of blood pressure leads to are duction in 35% of retinopathy, a 35% need for retinal laser, and 50% blindness [41].

Similarly, poor glycemic control status was the determinant factor for diabetic retinopathy. Diabetic patients who have poor glycemic control status were nearly 4.4 times more likely to develop diabetic retinopathy compared to those diabetic patients with good glycemic control status. The finding of this study is supported by the study conducted in China [36, 42]. The possible reason is that too much blood glucose in the blood may block the tiny blood vessels that nourish the retina. As a result, the eye attempts to grow new blood vessels, but these new blood vessels do not develop properly and can leak easily, which leads to vascular edema [43]. Living with high blood glucose is the trigger for retinal vascular structure abnormality. The endothelial cells’ malfunction owing to chronic exposure to high levels of glucose leads to endothelial cell malfunction. The resulting lesions include thickened capillary basement membrane, defects in the blood-retinal barrier, and pericyte loss [44].

Furthermore, living longer time with diabetes was a risk factor for diabetic retinopathy. Patients who have a longer duration of diabetic illness were nearly four times more likely to have diabetic retinopathy compared to those diabetic patients with a short duration of diabetic illness. This is supported by the study conducted in China [36, 45]. Patients with diabetes develop retinopathy within the early stages of the disease, but this does not affect the sight unless the patient is treated, it progresses and eventually affects the sight [46].

Lifestyle intervention is remarkably effective in the primary prevention of diabetes and HTN. The initial approach to the management of both diabetes and HTN must emphasize weight control, regular physical activity, and dietary modification [47]. Further actions such as health education on a meal plan, treatment adherence, and blood glucose monitoring may improve blood glucose status.

The study has important limitations. Firstly, the study was conducted on both the type 1 and 2 diabetic populations. Secondly, the authors used specific factors to see their effect on diabetic retinopathy. Thirdly, the incidence of DR was not included in this study. Therefore, we recommended that further research need to be carried out on type 1 and 2 diabetic population separately, including other important factors. Moreover, the incidence of diabetic retinopathy need to be investigated based on the population characteristics.

### Implication of the study

The study’s findings may result in a paradigm shift in the diabetic management process to reduce diabetic eye complications. As a result, health care providers, policymakers, and program planners could benefit from the study’s findings. The study has an implication for clinicians’ to provide important information about how effective a medical intervention to prevent the occurrence of diabetic retinopathy through early screening and treatment. The study also helps for decision and policy makers to plan and implement possible strategies to reduce the risks and progression of diabetic retinopathy.

## Conclusion

In this systematic and meta-analysis study, co-morbid HTN, poor glycemic control, and longer duration of diabetes illness were found to be the determinant factors of DR. Therefore, aggressive treatment of co-morbid HTN and blood glucose, and regular eye screening should be implemented to reduce the occurrence of DR among diabetic patients. In addition, healthcare workers should give due attention to those patients who have co-morbid HTN, poor glycemic control, and longer duration of diabetic illness.

## Supporting information

S1 File PRISMA checklist. (DOCX)

S2 File Data availability statement. (DOCX)

## Abbreviations

AOR: Adjusted Odds Ratio
CI: Confidence Interval
DR: Diabetic retinopathy
HbA1c: Glycated hemoglobin
HTN: Hypertension
OR: Odds Ratio
PRISMA: Preferred Reporting Item of Systematic Review and Meta-Analysis
SNNP: Southern Nations Nationalities and People

## References

[1] Wang W, Lo AC. Diabetic retinopathy: pathophysiology and treatments. International journal of molecular sciences. 2018;19(6):1816. doi: 10.3390/ijms19061816 29925789 PMC6032159

[2] He Z, King GL. Microvascular complications of diabetes. Endocrinology and Metabolism Clinics. 2004;33(1):215–38. doi: 10.1016/j.ecl.2003.12.003 15053904

[3] Paulus YM, Blumenkranz MS. Proliferative and nonproliferative diabetic retinopathy. American Academy of Opthamology. 2013.

[4] Ferris FL III, Patz A. Macular edema. A complication of diabetic retinopathy. Survey of ophthalmology. 1984;28:452–61. doi: 10.1016/0039-6257(84)90227-3 6379946

[5] Ciulla TA, Amador AG, Zinman B. Diabetic retinopathy and diabetic macular edema: pathophysiology, screening, and novel therapies. Diabetes care. 2003;26(9):2653–64. doi: 10.2337/diacare.26.9.2653 12941734

[6] Thomas R, Halim S, Gurudas S, Sivaprasad S, Owens D. IDF Diabetes Atlas: A review of studies utilising retinal photography on the global prevalence of diabetes related retinopathy between 2015 and 2018. Diabetes research and clinical practice. 2019;157:107840. doi: 10.1016/j.diabres.2019.107840 31733978

[7] Fong DS, Aiello L, Gardner TW, King GL, Blankenship G, Cavallerano JD, et al. Retinopathy in diabetes. Diabetes care. 2004;27(suppl_1):s84–s7. doi: 10.2337/diacare.27.2007.s84 14693935

[8] Teo ZL, Tham YC, Yu M, Chee ML, Rim TH, Cheung N, et al. Global Prevalence of Diabetic Retinopathy and Projection of Burden through 2045: Systematic Review and Meta-analysis. Ophthalmology. 2021;128(11):1580–91. doi: 10.1016/j.ophtha.2021.04.027 33940045

[9] Fite RO, Lake EA, Hanfore LK. Diabetic retinopathy in Ethiopia: a systematic review and meta-analysis. Diabetes & Metabolic Syndrome: Clinical Research & Reviews. 2019;13(3):1885–91. 10.1016/j.dsx.2019.04.016 31235110

[10] Tantigegn S, Ewunetie AA, Agazhe M, Aschale A, Gebrie M, Diress G, et al. Time to diabetic neuropathy and its predictors among adult type 2 diabetes mellitus patients in Amhara regional state Comprehensive Specialized Hospitals, Northwest Ethiopia, 2022: A retrospective follow up study. 2023;18(4):e0284568. 10.1371/journal.pone.0284568 PMC10146479 37115732

[11] Natalia L. Pathophysiology of Diabetic Retinopathy. In: Giuseppe Lo G, editor. Diabetic Eye Disease. Rijeka: IntechOpen; 2022. p. Ch. 1.

[12] Beaser RS, Turell WA, Howson A. Strategies to improve prevention and management in diabetic retinopathy: qualitative insights from a mixed-methods study. Diabetes Spectrum. 2018;31(1):65–74. doi: 10.2337/ds16-0043 29456428 PMC5813310

[13] Honasoge A, Nudleman E, Smith M, Rajagopal R. Emerging insights and interventions for diabetic retinopathy. Current diabetes reports. 2019;19(10):1–16. doi: 10.1007/s11892-019-1218-2 31506830 PMC7941754

[14] Ockrim Z, Yorston D. Managing diabetic retinopathy. Bmj. 2010;341. doi: 10.1136/bmj.c5400 20974661

[15] Page MJ, McKenzie JE, Bossuyt PM, Boutron I, Hoffmann TC, Mulrow CD, et al. The PRISMA 2020 statement: an updated guideline for reporting systematic reviews. Systematic reviews. 2021;10(1):1–11. 33781348 10.1186/s13643-021-01626-4 PMC8008539

[16] McPheeters ML, Kripalani S, Peterson NB, Idowu RT, Jerome RN, Potter SA, et al. Quality improvement interventions to address health disparities: closing the quality gap-revisiting the state of the science. Database of Abstracts of Reviews of Effects (DARE): Quality-assessed Reviews [Internet]. 2012. PMC4781280 24422952

[17] Yepes-Nuñez J, Urrutia G, Romero-Garcia M, Alonso-Fernandez S. The PRISMA 2020 statement: an updated guideline for reporting systematic reviews. Revista Espanola de Cardiologia (English ed). 2021;74(9):790–9. 34446261 10.1016/j.rec.2021.07.010

[18] Rücker G, Schwarzer G, Carpenter JR, Schumacher M. Undue reliance on I 2 in assessing heterogeneity may mislead. BMC medical research methodology. 2008;8(1):1–9. 19036172 10.1186/1471-2288-8-79 PMC2648991

[19] Borenstein M, Hedges LV, Higgins JP, Rothstein HR. A basic introduction to fixed‐effect and random‐effects models for meta‐analysis. Research synthesis methods. 2010;1(2):97–111. doi: 10.1002/jrsm.12 26061376

[20] Begg CB, Mazumdar M. Operating characteristics of a rank correlation test for publication bias. Biometrics. 1994:1088–101. 7786990

[21] Tilahun M, Gobena T, Dereje D, Welde M, Yideg G. Prevalence of Diabetic retinopathy and its associated factors among diabetic patients at Debre Markos referral hospital, Northwest Ethiopia, 2019: hospital-based cross-sectional study. Diabetes, Metabolic Syndrome and Obesity: Targets and Therapy. 2020;13:2179. doi: 10.2147/DMSO.S260694 32636659 PMC7328291

[22] Alemayehu HB, Tegegn MT, Tilahun MM. Prevalence and associated factors of visual impairment among adult diabetic patients visiting Adare General Hospital, Hawassa, South Ethiopia, 2022. Plos one. 2022;17(10):e0276194. doi: 10.1371/journal.pone.0276194 36227943 PMC9560493

[23] Getasew Alemu Mersha YAA, Mikias Mered Tilahun, TarkegnCheklie Zeleke, Asamere Tsegaw Woredekal. Prevalence of Diabetic Retinopathy and Associated Risk Factors among Adult Diabetes Attending at Debre Tabor General Hospital, Northwest Ethiopia. Journal of Diabetes & Metabolism. May 25, 2021;14(4).

[24] Seid K, Tesfaye T, Belay A, Mohammed H. Determinants of diabetic retinopathy in Tikur Anbessa Hospital, Ethiopia: a case–control study. Clinical Diabetes and Endocrinology. 2021;7(1):1–9. 34325741 10.1186/s40842-021-00128-5 PMC8323205

[25] Garoma D, Merga H, Hiko D. Determinants of diabetic retinopathy in Southwest Ethiopia: a facility-based case-control study. BMC Public Health. 2020;20(1):1–9. 32295571 10.1186/s12889-020-08652-2 PMC7161237

[26] Tsion Shiberu FA, & Abdissa Boka. Prevalence and factors associated with diabetes retinopathy among type 2 diabetes patients at Tikur Anbesa specialized hospital Addis Ababa, Ethiopia, 2018. 2018.

[27] Ejigu T, Tsegaw A. Prevalence of diabetic retinopathy and risk factors among diabetic patients at University of Gondar Tertiary Eye Care and Training Center, North-West Ethiopia. Middle East African Journal of Ophthalmology. 2021;28(2):71. doi: 10.4103/meajo.meajo_24_21 34759663 PMC8547671

[28] Chisha Y, Terefe W, Assefa H. Incidence and factors associated with diabetic retinopathy among diabetic patients at arbaminch general hospital, gamo gofa Zone (longitudinal follow up data analysis). Journal of Diabetology. 2017;8(1):1.

[29] Azeze TK, Sisay MM, Zeleke EG. Incidence of diabetes retinopathy and determinants of time to diabetes retinopathy among diabetes patients at Tikur Anbessa Hospital, Ethiopia: a retrospective follow up study. BMC research notes. 2018;11(1):1–6. 30068385 10.1186/s13104-018-3660-7 PMC6071390

[30] Takele MB, Boneya DJ, Alemu HA, Tsegaye TB, Birhanu MY, Alemu S, et al. Retinopathy among Adult Diabetics and Its Predictors in Northwest Ethiopia. Journal of Diabetes Research. 2022;2022. doi: 10.1155/2022/1362144 35211627 PMC8863468

[31] Gelcho GN, Gari FS. Time to Diabetic Retinopathy and Its Risk Factors among Diabetes Mellitus Patients in Jimma University Medical Center, Jimma, Southwest Ethiopia. Ethiopian Journal of Health Sciences. 2022;32(5):937–46. doi: 10.4314/ejhs.v32i5.9 36262700 PMC9554771

[32] Aberra T, Feleke Y, Tarekegn G, Bikila D, Melesse M. Prevalence and associated factors of diabetic nephropathy at Tikur Anbessa Comprehensive Specialized University Hospital, Addis Ababa, Ethiopia. African Journal of Nephrology. 2022;25(1):35–45.

[33] Debele GR, Kanfe SG, Weldesenbet AB, Ayana GM, Jifar WW, Raru TB. Incidence of diabetic retinopathy and its predictors among newly diagnosed type 1 and type 2 diabetic patients: a retrospective follow-up study at tertiary health-care setting of Ethiopia. Diabetes, Metabolic Syndrome and Obesity: Targets and Therapy. 2021;14:1305. doi: 10.2147/DMSO.S300373 33790598 PMC7997545

[34] Alemu S, Dessie A, Tsegaw A, Patterson CC, Parry EH, Phillips DI, et al. Retinopathy in type 1 diabetes mellitus: Major differences between rural and urban dwellers in northwest Ethiopia. Diabetes research and clinical practice. 2015;109(1):191–8. doi: 10.1016/j.diabres.2015.04.010 25944537

[35] Fekadu Abera YT, & Aklil Hailu. Prevalence of diabetic retinopathy and its associated factors among type two diabetes mellitus patients in Hiwot Fana Specialized University Hospital, Harar, Eastern Ethiopia, 2021. 2021.

[36] Zhang G, Chen H, Chen W, Zhang M. Prevalence and risk factors for diabetic retinopathy in China: a multi-hospital-based cross-sectional study. British Journal of Ophthalmology. 2017;101(12):1591–5. doi: 10.1136/bjophthalmol-2017-310316 28855195 PMC5754882

[37] Liu L, Quang ND, Banu R, Kumar H, Tham Y-C, Cheng C-Y, et al. Hypertension, blood pressure control and diabetic retinopathy in a large population-based study. PLoS One. 2020;15(3):e0229665. doi: 10.1371/journal.pone.0229665 32134944 PMC7058315

[38] Grosso A, Veglio F, Porta M, Grignolo F, Wong T. Hypertensive retinopathy revisited: some answers, more questions. British Journal of Ophthalmology. 2005;89(12):1646–54. doi: 10.1136/bjo.2005.072546 16299149 PMC1772998

[39] Wong TY, Mitchell P. Hypertensive retinopathy. New England Journal of Medicine. 2004;351(22):2310–7. doi: 10.1056/NEJMra032865 15564546

[40] Walsh JB. Hypertensive retinopathy: description, classification, and prognosis. Ophthalmology. 1982;89(10):1127–31. 7155523

[41] Klein R, Knudtson MD, Lee KE, Gangnon R, Klein BE. The Wisconsin Epidemiologic Study of Diabetic Retinopathy XXII: the twenty-five-year progression of retinopathy in persons with type 1 diabetes. Ophthalmology. 2008;115(11):1859–68. doi: 10.1016/j.ophtha.2008.08.023 19068374 PMC2761813

[42] Song P, Yu J, Chan KY, Theodoratou E, Rudan I. Prevalence, risk factors and burden of diabetic retinopathy in China: a systematic review and meta-analysis. Journal of global health. 2018;8(1). doi: 10.7189/jogh.08.010803 29899983 PMC5997368

[43] Shin ES, Sorenson CM, Sheibani N. Diabetes and retinal vascular dysfunction. Journal of ophthalmic & vision research. 2014;9(3):362. doi: 10.4103/2008-322X.143378 25667739 PMC4307665

[44] Gillow J, Gibson J, Dodson P. Hypertension and diabetic retinopathy—what’s the story? British journal of ophthalmology. 1999;83(9):1083–7. doi: 10.1136/bjo.83.9.1083 10460781 PMC1723193

[45] Zoungas S, Woodward M, Li Q, Cooper ME, Hamet P, Harrap S, et al. Impact of age, age at diagnosis and duration of diabetes on the risk of macrovascular and microvascular complications and death in type 2 diabetes. Diabetologia. 2014;57(12):2465–74. doi: 10.1007/s00125-014-3369-7 25226881

[46] Klein R, Klein BE. Vision disorders in diabetes. Diabetes in America. 1995;1:293.

[47] Long AN, Dagogo‐Jack S. Comorbidities of diabetes and hypertension: mechanisms and approach to target organ protection. The journal of clinical hypertension. 2011;13(4):244–51. doi: 10.1111/j.1751-7176.2011.00434.x 21466619 PMC3746062

