# Supplementary material for: Determinants of diabetic retinopathy in Ethiopia: A systematic review and meta-analysis
Source: PLoS One. 2023 Jun 8;18(6):e0286627. doi: 10.1371/journal.pone.0286627 (PMC10249865; doi:10.1371/journal.pone.0286627)
Supplement: S1 File — (DOCX) [file pone.0286627.s001.docx]

| **Section and Topic** | **Item #** | **Checklist item** | **Location where item is reported** |
| --- | --- | --- | --- |
| **TITLE** | | |  |
| Title | 1 | **Determinants of Diabetic Retinopathy in Ethiopia: A systematic review and meta-analysis** | 1 |
| **ABSTRACT** | | |  |
| Abstract | 2 | **Introduction**  Diabetic retinopathy (DR) is the primary retinal vascular complication of diabetes mellitus and a leading cause of visual impairment and blindness. It affects the global diabetic population. In Ethiopia, about one-fifth of diabetic patients were affected by DR, but little is known about the determinants of DR. Therefore, we aimed to identify the risk factors for DR among diabetic patients.  **Methods**: We have accessed previous studies through an electronic web-based search strategy using PubMed, Google (Scholar), the Web of Science, and the Cochrane Library with a combination of search terms. The quality of each included article was assessed using the Newcastle Ottawa Assessment Scale. All statistical analyses were carried out using stata version 14 software. The odds ratios of risk factors were pooled using a fixed-effect meta-analysis. Heterogeneity was assessed using Cochrane Q statistics and I-Square (I^2^). Furthermore, publication bias was detected based on the graphic asymmetry test of the funnel plot and/or Egger’s test (p< 0.05).  **Results:** The search strategy retrieved 1182 articles. After the removal of duplicate articles, 249 articles remained. Following further screening, about 18 articles were assessed for eligibility, of which three articles were excluded because of reporting without the outcome of interest, poor quality, and not full text. Finally, fifteen studies were reviewed for the final analysis. Co-morbid hypertension (HTN) (AOR 2.04, 95%CI: 1.07, 3.89), poor glycemic control (AOR=4.36, 95%CI: 1.47, 12.90), and duration of diabetes illness (AOR =3.83, 95%CI: 1.17, 12.55) were found to be confirmed associated factors of diabetic retinopathy.  **Conclusion**: In this study, co-morbid HTN, poor glycemic control, and longer duration of diabetes illness were found to be the determinant factors of DR. Aggressive treatment of co-morbid HTN and blood glucose, and regular eye screening should be implemented to reduce the occurrence of DR among diabetic patients.  **Keywords**: diabetes mellitus, diabetic retinopathy, determinants, risk factors, Ethiopia. | 2 |
| **INTRODUCTION** | | |  |
| Rationale | 3 | Diabetic retinopathy (DR) is the primary retinal vascular complication of diabetes mellitus and a leading cause of visual impairment and blindness. Clinically, diabetic retinopathy is classified as non-proliferative and proliferative diabetic retinopathy. Non-proliferative diabetic retinopathy (NPDR) is the earliest and the asymptomatic stage whereas proliferative diabetic retinopathy (PDR) is the advanced stage of diabetic retinopathy characterized by neovascularization. In the PDM stage, the patient experiences severe vision impairment when the new abnormal blood vessel bleeds to the vitreous (vitreous hemorrhage) or retinal detachment  It affects the global diabetic population. In Ethiopia, about one-fifth of diabetic patients were affected by DR, but little is known about the determinant factors. Therefore, risk stratification and selective early intervention for high-risk patients need to be given attention. In Ethiopia, there were different research articles reporting on the determinant factors of diabetic retinopathy, but their findings were inconsistent across the studies. Therefore, this study aimed to identify the determinant factors of DR among diabetic patients. | 3 |
| Objectives | 4 | To identify factors associated with diabetic retinopathy among diabetic patients in Ethiopia |  |
| **METHODS** | | |  |
| Eligibility criteria | 5 | \| **Criteria** \| **Inclusion criteria** \| **Exclusion criteria** \| \| --- \| --- \| --- \| \| Participants \| people with diabetic retinopathy  age ≥18 years \| Population with no outcome interest \| \| Study setting \| hospital or health facility \| Community-based study \| \| Design \| observational study designs  (cross-sectional, cohort, and case-control) \|  \| \| Publication status \| both published and unpublished studies \| Qualitative studies  conference papers  articles with no full text \| \| Language of publication \| English \| Languages other than the English language \| \| Country \| Ethiopia  different regions of the country \|  \| \| Publication year \| No restriction \|  \| | 6 |
| Information sources | 6 | We searched on PubMed, Google (for grey literature), Google Scholar, Web of Science, and Cochrane Library databases for studies reporting diabetic retinopathy. | 4 |
| Search strategy | 7 | Our comprehensive search strategies were carried out using controlled vocabularies (MeSH terms). Using the MeSH database, the synonyms of diabetic retinopathy were identified. Then, the search string was established using the databases. Articles were searched by title (Ti), abstract (Ab), full text, or all these categories. Modification of the search strategy was made by limiters such as publication year and country. Boolean logic operators like “AND” and “OR” were used to combine searching terms. Two reviewers independently searched and screened articles by title, abstract, and full text. The disagreements between the reviewers were resolved by discussion. | 4 |
| Selection process | 8 | Eligible research articles were screened by their title (Ti), Abstract (Ab), and full-text. Two reviewers independently reviewed the included articles. | 4 |
| Data collection process | 9 | The data were extracted by data abstraction format using the Microsoft excel spreadsheet. The format was developed by two reviewers and piloted for its clarity, aim, consistency, and depth of the contents. Simple and consistent codes of response were used. Then the reviewers independently reviewed and extract data from each eligible study. The information such as authors, publication year, region of the study, design, methodological quality, population, study setting, sample size, method of data collection, statistical analysis, and funding source were extracted from the studies. | 6 |
| Data items | 10a | Articles that have clearly defined outcome variables were included | 6 |
|  | 10b | The variables that have direct effect for the occurrence of Diabetic retinopathy were identified. | 6 |
| Study risk of bias assessment | 11 | Articles were assessed for quality score using the New Castle Ottawa Scale adapted from cross-sectional, cohort and case-control quality assessment tool; a score of ≥ 7 out of 10 was considered a high-quality score. Two authors assessed the quality of each paper. The reviewers compared the quality of the appraisal scores and resolved inconsistencies prior to calculating the final appraisal score. | 6 |
| Effect measures | 12 | The odds ratio (or), logor, and standard error or (SeOr) were used presentation of results. | 6 |
| Synthesis methods | 13a | Both qualitative synthesis and quantitative analysis were employed. | 4 |
|  | 13b | The crud odds ratio(COR) or adjusted odds ratio(AOR) were used for data presentation or evidence synthesis. | 4 |
|  | 13c | PRISMA flow chart, forest plot, and funnel plot were used to present visually displayed data. | 5 |
|  | 13d | The Cochrane Q statistics and (**I**^2^) were used to assess the heterogeneity status of the included studies. | 7 |
|  | 13e | A fixed effect model was used for analysis. | 7 |
|  | 13f | The quantitative synthesis was employed | 7 |
| Reporting bias assessment | 14 | Begg’s and/or Egger’s test was computed to detect publication bias. | 7 |
| Certainty assessment | 15 | The pooled summary effect size of the study was estimated | 7 |
| **RESULTS** | | |  |
| Study selection | 16a | The search strategy retrieved 1285 articles. After the removal of duplicate articles, 249 articles remained. Following the additional screening, eighteen articles were evaluated for eligibility, with three being excluded due to being incomplete, of poor quality, or not in full text (**Fig1**). Finally, fifteen studies were reviewed for co-morbid HTN ([20-34](#_ENREF_20)) (**Table 2**), six studies for poor glycemic control ([20](#_ENREF_20), [21](#_ENREF_21), [23](#_ENREF_23), [24](#_ENREF_24), [27](#_ENREF_27), [31](#_ENREF_31)) (**Table 3**), and five studies for the duration of diabetic illness([20](#_ENREF_20), [21](#_ENREF_21), [24](#_ENREF_24), [26](#_ENREF_26), [30](#_ENREF_30)) (**Table 4**). | 8 |
|  | 16b | Studies were excluded because of outcome interest and not full text articles. | 7 |
| Study characteristics | 17 | Of the total studies; five were conducted in the Amhara region ([20](#_ENREF_20), [22](#_ENREF_22), [26](#_ENREF_26), [29](#_ENREF_29), [33](#_ENREF_33)), five in Addis Ababa ([23](#_ENREF_23), [25](#_ENREF_25), [28](#_ENREF_28), [31](#_ENREF_31), [34](#_ENREF_34)), three in the Oromia region ([24](#_ENREF_24), [30](#_ENREF_30), [32](#_ENREF_32)), and two in South Nations and Nationalities people (SNNP) of Ethiopia ([21](#_ENREF_21), [27](#_ENREF_27)). All the studies were published between the years 2015 and 2022. Regarding the study design, eight studies were cross-sectional ([20-22](#_ENREF_20), [25](#_ENREF_25), [26](#_ENREF_26), [31](#_ENREF_31), [33](#_ENREF_33), [34](#_ENREF_34)), five studies were cohort ([27-30](#_ENREF_27), [32](#_ENREF_32)), and two studies were case-control ([23](#_ENREF_23), [24](#_ENREF_24)). | 7 |
| Risk of bias in studies | 18 | In terms of their quality status, all the included studies have high quality | 8 |
| Results of individual studies | 19 | \| Author/year \| Study design \| Region of  the study \| data collection  technique \| Funding Source \| Factors \| AOR \| 95% CI \| overall quality score \| \| --- \| --- \| --- \| --- \| --- \| --- \| --- \| --- \| --- \| \| Tilahun M, et al/2020([20](#_ENREF_20)) \| Cross-sectional \| Amhara \| Interview &  patient review \| not funded \| Co-HTN \| 3.39 \| 1.64-7.02 \| 8.5 \| \| Alemayehu HB, et al./2022([21](#_ENREF_21)) \| Cross-sectional \| SNNP \| Interview & patient chart review \| not reported \| Co-HTN \| 1.43 \| 0.72-2.86 \| 7.4 \| \| Mersha GA, et al./2021([22](#_ENREF_22)) \| Cross-sectional \| Amhara \| interview & chart review \| not reported \| Co-HTN \| 1.67 \| 0.66-4.20 \| 7 \| \| Seid K, et al./2021([23](#_ENREF_23)) \| case-control \| Addis Ababa \| interview & chart review \| Jimma University, Institute of Health \| Co-HTN \| 12.3 \| 6.95-21.8 \| 7.5 \| \| Garoma D etal./2020([24](#_ENREF_24)) \| Case-control \| Oromia \| interview & chart review  Ocular exam \| Jimma University, Institute of Health \| Co-HTN \| 3.38 \| 1.29-9.05 \| 7.0 \| \| Shibru T, et al./2018([25](#_ENREF_25)) \| Cross-sectional \| Addis Ababa \| interview & chart review  Ocular exam \| Not reported \| Co-HTN \| 2.556 \| 1.014-6.447 \| 9.0 \| \| Ejigu T, et al/2021([26](#_ENREF_26)) \| Cross-sectional \| Amhara \| interview & chart review  Ocular exam \| Not reported \| Co-HTN \| 2.65 \| 1.02-6.87 \| 8.0 \| \| Chisha Y, etal./2017([27](#_ENREF_27)) \| Cohort \| SNNP \| Record review \| Mekelle University \| Co-HTN \| 4.1 \| 1.76-9.44 \| 7.5 \| \| Azeze TK, et al./2018([28](#_ENREF_28)) \| Cohort \| Addis Ababa \| Record review \| self sponsored \| Co-HTN \| 1.51 \| 0.48-4.74 \| 8.5 \| \| Takele MB, et al./2022([29](#_ENREF_29)) \| Cohort \| Amhara \| Record review \| Amhara regional state \| Co-HTN \| 1.68 \| 1.14-2.50 \| 8.4 \| \| Gelcho GN, et al./2022([30](#_ENREF_30)) \| Cohort \| Oromia \| Record review \| Not Funded \| Co-HTN \| 2.32 \| 1.12-4.39 \| 7.6 \| \| Aberra T, et al./2022([31](#_ENREF_31)) \| Cross-sectional \| Addis Ababa \| Interview  Record review \| Not reported \| Co-HTN \| 1.37 \| 0.865-2.169 \| 8.5 \| \| Debele GR,et al./2021([32](#_ENREF_32)) \| Cohort \| Oromia \| Record review \| University of Gondar \| Co-HTN \| 0.54 \| 0.35-0.82 \| 8.0 \| \| Alemu S, et al./2015([33](#_ENREF_33)) \| Cross-sectional \| Amhara \| Interview & record review \| not reported \| Co-HTN \| 5.2 \| 2.5-10.20 \| 7.5 \| \| Abera F, et al./2021([34](#_ENREF_34)) \| Cross-sectional \| Addis Ababa \| interview & chart review  ocular exam \| Not reported \| Co-HTN \| 8.63 \| 2.51-29.75 \| 7.0 \| | 9 |
| Results of syntheses | 20a | The risk of bias was assed using Begg’s and/or Egger’s test. | 8 |
|  | 20b | **Determinants of diabetic retinopathy**  **Co-morbid Hypertension**  In this systematic review and meta-analysis, co-morbid HTN is found to be the determinant factor for diabetic retinopathy. Diabetic patients who have co-morbid HTN are 2.04 times more likely to have diabetic retinopathy compared to those diabetic patients with no co-morbid HTN (AOR 2.04, 95%CI: 1.07, 3.89) (**Fig** **2**).  **Duration of diabetic illness**  Longer duration of diabetic illness is found to be the risk factor for the development of diabetic retinopathy. Patients who had a longer duration of diabetic illness were nearly four times more likely to have diabetic retinopathy compared to those diabetic patients with a shorter duration of diabetic illness (AOR =3.83, 95%CI: 1.17, 12.55, I^2^=0.0% and p-value = 0.999) (**Fig** 6).  **Poor glycemic control**  In this study, poor glycemic control was found to be the determinant factor for diabetic retinopathy. Diabetic patients with poor glycemic control status were 4.36 times more likely to have diabetic retinopathy compared to those diabetic patients with good glycemic control status (AOR=4.36, 95%CI: 1.47, 12.90) (**Fig.** 4). | 11 |
|  | 20c | The overall heterogeneity test (**I^2^**) on the effect of co-morbid HTN was 0.0% with a p-value < 0.946, using a random effect model to adjust observed variability. This indicates there is no variability across the studies. | 12 |
|  | 20d | Regarding the publication bias, the graphic asymmetry test of the funnel plot which shows a symmetrical distribution (**Fig** 3), and Egger’s test p-value= 0.181, indicating that there is no publication bias. | 10 |
| Reporting biases | 21 | This study hasn’t publication bias as shown by the inverted funnel plot which symmetrically distributed (**Fig** 3). Begg's test and Egger’s test were done with p >0.494 which showed that the absence of publication bias. | 12 |
| Certainty of evidence | 22 | In this systematic and meta-analysis study, co-morbid HTN, poor glycemic control, and longer duration of diabetes illness were found to be the determinant factors of DR. | 15 |
| **DISCUSSION** | | |  |
| Discussion | 23a | The pooled effect of this study depicts that diabetic patients who have co-morbid HTN were 2.04 times more likely to have diabetic retinopathy compared to those diabetic patients with no co-morbid HTN. The finding of this study is supported by large-scale studies in China ([35](#_ENREF_35)), and (Chinese, Malay, and India) ([36](#_ENREF_36)). This is the fact that HTN has a direct impact on retinal blood vessels. It damages the retinal vascular structures([37](#_ENREF_37)). The elevated blood pressure is transferred directly to the vessels, which initially constrict, but a further increase in BP overcomes this compensatory tone, and damage to the muscle layer and endothelium ensues ([38](#_ENREF_38)). This results in retinal edema, cotton wool spots, hemorrhage, and disc edema ([39](#_ENREF_39)). Evidence showed that tight blood pressure control in the diabetic population reduces the incidence of sight-threatening retinopathy with a favorable impact on the lives of diabetic patients. A decrease in every 10 mmHg of blood pressure leads to a reduction in 35% of retinopathy, a 35% need for retinal laser, and 50% blindness ([40](#_ENREF_40)). laser, and 50% blindness ([40](#_ENREF_40)).  Similarly, poor glycemic control status was the determinant factor for diabetic retinopathy. Diabetic patients who have poor glycemic control status were nearly 4.4 times more likely to develop diabetic retinopathy compared to those diabetic patients with good glycemic control status. The finding of this study is supported by the study conducted in China ([35](#_ENREF_35), [41](#_ENREF_41)). The possible reason is that too much blood glucose in the blood may block the tiny blood vessels that nourish the retina. As a result, the eye attempts to grow new blood vessels, but these new blood vessels don’t develop properly and can leak easily, which leads to vascular edema ([42](#_ENREF_42)). Living with high blood glucose is the trigger for retinal vascular structure abnormality. The endothelial cells’ malfunction owing to chronic exposure to high levels of glucose leads to endothelial cell malfunction. The resulting lesions include thickened capillary basement membrane, defects in the blood-retinal barrier, and pericyte loss ([43](#_ENREF_43)).  Furthermore, living longer time with diabetes was a risk factor for diabetic retinopathy. Patients who have a longer duration of diabetic illness were nearly four times more likely to have diabetic retinopathy compared to those diabetic patients with a short duration of diabetic illness. This is supported by the study conducted in China ([35](#_ENREF_35), [44](#_ENREF_44)). Patients with diabetes develop retinopathy within the early stages of the disease, but this does not affect the sight unless the patient is treated, it progresses and eventually affects the sight ([45](#_ENREF_45)). | 13 |
|  | 23b | The study has important limitations. Firstly, the study was conducted on both the type I and II diabetic populations. It has its impact to act up on the problem. Secondly, the authors used specific factors to see their effect on diabetic retinopathy.  Thirdly, the incidence of DR was not included in this study. Therefore, we recommended that further research need to be carried out on type I and II diabetic population separately, including other important factors. Moreover, the incidence of diabetic retinopathy need to be investigated based on the population characteristics. | 14 |
|  | 23c | The study has an implication for policymakers and clinicians to plan and implement possible interventions to prevent the occurrence and severe outcome of diabetic retinopathy. | 14 |
|  | 23d | Therefore, aggressive treatment of co-morbid HTN and blood glucose, and regular eye screening should be implemented to reduce the occurrence of DR among diabetic patients. In addition, healthcare workers should give due attention to those patients who have co-morbid HTN, poor glycemic control, and longer duration of diabetic illness. | 15 |
| **OTHER INFORMATION** | | |  |
| Registration and protocol | 24a | The review protocol has been registered in the international prospective register of systematic reviews (PROSPERO) with registration number PROSPERO: CRD42023416724. |  |
|  | 24b | The review protocol can be accessed via online databases. |  |
|  | 24c | Further amendments may /not needed. |  |
| Support | 25 | The Authors did not receive any fund for this particular study. |  |
| Competing interests | 26 | There is no competing of interest. |  |
| Availability of data, code and other materials | 27 | The data extracted were analysed and included in the result. |  |

*From:* Page MJ, McKenzie JE, Bossuyt PM, Boutron I, Hoffmann TC, Mulrow CD, et al. The PRISMA 2020 statement: an updated guideline for reporting systematic reviews. BMJ 2021;372:n71.doi: 10.1136/bmj.n71

For more information, visit:<http://www.prisma-statement.org/>
