# Supplementary material for: Determinants of diabetic retinopathy in Ethiopia: A systematic review and meta-analysis
Source: PLoS One. 2023 Jun 8;18(6):e0286627. doi: 10.1371/journal.pone.0286627 (PMC10249865; doi:10.1371/journal.pone.0286627)
Supplement: S2 File — (DOCX) [file pone.0286627.s002.docx]

**Supportive information on data availability**

**co-morbid Hypertension**

| **S. no** | **Author (s)** | **year of public**  **cation** | **country** | **Region** | **Study**  **popu**  **lation** | **study**  **design** | **Data collection**  **technique** | **Factors** | **AOR** | **logOR** | **LBCI** | **log**  **LBCI** | **UBCI** | **Log**  **UBCI** | **SeLog**  **OR** | **Funding**  **source** | **Quality appraisal score** |
| --- | --- | --- | --- | --- | --- | --- | --- | --- | --- | --- | --- | --- | --- | --- | --- | --- | --- |
| 1 | Tilahun M, et al/([20](#_ENREF_20)) | 2020 | Ethiopia | Amhara | diabetic patients | Cross-sectional | Interview &  patient review | Co-HTN | 3.39 | 1.22083 | 1.64 | 0.494696 | 7.02 | 1.948763 | 1.424986 | not funded | 8.5 |
| 2 | Alemayehu HB, et al./([21](#_ENREF_21)) | 2022 | Ethiopia | SNNP | diabetic patients | Cross-sectional | Interview & patient chart review | Co-HTN | 1.43 | 0.357674 | 0.72 | -0.3285 | 2.86 | 1.050822 | 1.351739 | not reported | 7.4 |
| 3 | Mersha GA, et al./([22](#_ENREF_22)) | 2021 | Ethiopia | Amhara | diabetic patients | Cross-sectional | interview & chart review |  | 1.67 | 0.512824 | 0.66 | -0.41552 | 4.2 | 1.435085 | 1.813588 | not reported | 7 |
| 4 | Seid K, et al./1([23](#_ENREF_23)) | 2021 | Ethiopia | Addis Ababa | diabetic patients | case-control | interview & chart review | Co-HTN | 12.3 | 2.509599 | 6.95 | 1.938742 | 21.8 | 3.08191 | 1.120305 | Jimma University, Institute of Health | 7.5 |
| 5 | Garoma D etal./([24](#_ENREF_24)) | 2020 | Ethiopia | Oromia | diabetic patients | Case-control | interview & chart review  Ocular exam | Co-HTN | 3.38 | 1.217876 | 1.26 | 0.231112 | 9.05 | 2.202765 | 1.93222 | Jimma University, Institute of Health | 7.0 |
| 6 | Shibru T, et al./([25](#_ENREF_25)) | 2018 | Ethiopia | Addis Ababa | diabetic patients | Cross-sectional | interview & chart review  Ocular exam | Co-HTN | 2.556 | 0.938444 | 1.014 | 0.013903 | 6.447 | 1.863615 | 1.812718 | Not reported | 9.0 |
| 7 | Ejigu T, et al/([26](#_ENREF_26)) | 2021 | Ethiopia | Amhara | diabetic patients | Cross-sectional | interview & chart review  Ocular exam | Co-HTN | 2.65 | 0.97456 | 1.02 | 0.019803 | 6.87 | 1.927164 | 1.869214 | Not reported | 8.0 |
| 8 | Chisha Y, etal./([27](#_ENREF_27)) | 2017 | Ethiopia | SNNP | diabetic patients | Cohort | Record review | Co-HTN | 4.1 | 1.410987 | 1.76 | 0.565314 | 9.44 | 2.244956 | 1.646049 | Mekelle University | 7.5 |
| 9 | Azeze TK, et al./([28](#_ENREF_28)) | 2018 | Ethiopia | Addis Ababa | diabetic patients | Cohort | Record review | Co-HTN | 1.51 | 0.41211 | 0.48 | -0.73397 | 4.74 | 1.556037 | 2.244206 | self sponsored | 8.5 |
| 10 | Takele MB, et al./([29](#_ENREF_29)) | 2022 | Ethiopia | Amhara | diabetic patients | Cohort | Record review | Co-HTN | 1.68 | 0.518794 | 1.14 | 0.131028 | 2.5 | 0.916291 | 0.769557 | Amhara regional state | 8.4 |
| 11 | Gelcho GN, et al./([30](#_ENREF_30)) | 2022 | Ethiopia | Oromia | diabetic patients | Cohort | Record review | Co-HTN | 2.32 | 0.841567 | 1.12 | 0.113329 | 4.39 | 1.479329 | 1.338681 | Not Funded | 7.6 |
| 12 | Aberra T, et al./([31](#_ENREF_31)) | 2022 | Ethiopia | Addis Ababa | diabetic patients | Cross-sectional | Interview  Record review | Co-HTN | 1.37 | 0.314811 | 0.865 | -0.14503 | 2.169 | 0.774266 | 0.900906 | Not reported | 8.5 |
| 13 | Debele GR,et al./([32](#_ENREF_32)) | 2021 | Ethiopia | Oromia | diabetic patients | Cohort | Record review | Co-HTN | 0.54 | -0.61619 | 0.35 | -1.04982 | 0.82 | -0.19845 | 0.834344 | University of Gondar | 8.0 |
| 14 | Alemu S, et al./([33](#_ENREF_33)) | 2015 | Ethiopia | Amhara | diabetic patients | Cross-sectional | Interview & record review | Co-HTN | 5.2 | 1.648659 | 2.5 | 0.916291 | 10.2 | 2.322388 | 1.377975 | not reported | 7.5 |
| 15 | Abera F, et al./([34](#_ENREF_34)) | 2021 | Ethiopia | Addis Ababa | diabetic patients | Cross-sectional | interview & chart review  ocular exam | Co-HTN | 8.63 | 2.155245 | 2.51 | 0.920283 | 29.75 | 3.392829 | 2.423095 | Not reported | 7.0 |

**Notes**; AOR; Adjusted odds ratio, CI; Confidence Interval, Co-HTN: Co-morbid Hypertension, SNNP: Southern Nations, Nationalities and Peoples’

**On poor glycemic control**

| **S. no** | **Author (s)** | **year of public**  **cation** | **country** | **Region** | **Study**  **popu**  **lation** | **study**  **design** | **Data collection**  **technique** | **Factors** | **AOR** | **logOR** | **LBCI** | **log**  **LBCI** | **UBCI** | **Log**  **UBCI** | **SeLog**  **OR** | **Funding**  **source** | **Quality appraisal score** |
| --- | --- | --- | --- | --- | --- | --- | --- | --- | --- | --- | --- | --- | --- | --- | --- | --- | --- |
| 1 | Tilahun M,etal/  ([20](#_ENREF_20)) | 2020 | Ethiopia | Amhara | diabetic patients | Crosssectional | Interview &  patient review | PGC | 4.58 | 1.521699 | 1.86 | 0.620576 | 11.31 | 2.425687 | 1.769009 | not  funded | 8.5 |
| 2 | Alemayehu HB, etal./([21](#_ENREF_21)) | 2022 | Ethiopia | SNNP | diabetic patients | Crosssectional | Interview & patient chart review | PGC | 4.34 | 1.467874 | 2.26 | 0.815365 | 8.34 | 2.121063 | 1.279584 | not reported | 7.4 |
| 3 | Mersha GA, etal./([22](#_ENREF_22)) | 2021 | Ethiopia | Amhara | diabetic patients | Cros sectional | interview & chart review | PGC | 3.2 | 1.163151 | 1.5 | 0.405465 | 6.7 | 1.902108 | 1.46671 | not reported | 7 |
| 4 | Seid K, etal./([23](#_ENREF_23)) | 2021 | Ethiopia | Addis Ababa | diabetic patients | case control | interview & chart review | PGC | 10.7 | 2.370244 | 6.17 | 1.819699 | 18.58 | 2.922086 | 1.080339 | Jimma University | 7.5 |
| 5 | Garoma D et al./([24](#_ENREF_24)) | 2020 | Ethiopia | Oromia | diabetic patients | Case-control | interview & chart review  Ocular exam | PGC | 9.08 | 2.206074 | 3.7 | 1.308333 | 22.29 | 3.104138 | 1.759889 | Jimma University | 7.0 |
| 6 | Aberra T, et al./([31](#_ENREF_31)) | 2022 | Ethiopia | Addis Ababa | diabetic patients | Cross-sectional | Interview  Record review | PGC | 1.23 | 0.207014 | 0.667 | -0.40497 | 2.276 | 0.82242 | 1.202837 | Not reported | 8.5 |

**Notes;** AOR; Adjusted odds ratio, CI; Confidence Interval, PGC: Poor glycemic control, SNNP: Southern Nations, Nationalities and Peoples’

**duration of diabetic illness**

| **S. no** | **Author (s)** | **year of public**  **cation** | **country** | **Region** | **Study**  **popu**  **lation** | **study**  **design** | **Data collection**  **technique** | **Factors** | **AOR** | **logOR** | **LBCI** | **log**  **LBCI** | **UBCI** | **Log**  **UBCI** | **SeLog**  **OR** | **Funding**  **source** | **Quality appraisal score** |
| --- | --- | --- | --- | --- | --- | --- | --- | --- | --- | --- | --- | --- | --- | --- | --- | --- | --- |
| 1 | Tilahun M,etal/([20](#_ENREF_20)) | 2020 | Ethiopia | Amhara | diabetic patients | Crosssectional | Interview &  patient review | Duration Diabetic illness | 3.91 | 1.363537 | 1.86 | 0.620576 | 8.23 | 2.107786 | 1.457465 | not funded | 8.5 |
| 2 | Alemayehu HB,etal./([21](#_ENREF_21)) | 2022 | Ethiopia | SNNP | diabetic patients | Crosssectional | Interview & patient chart review | Duration Diabetic illness | 4.78 | 1.564441 | 2.11 | 0.746688 | 10.83 | 2.38232 | 1.602919 | not reported | 7.4 |
| 3 | Garoma D etal./([24](#_ENREF_24)) | 2020 | Ethiopia | Oromia | diabetic patients | Case-control | interview & chart review  Ocular exam | Duration Diabetic illness | 4.38 | 1.477049 | 2.65 | 0.97456 | 7.22 | 1.976855 | 0.982249 | Jimma University | 7.0 |
| 4 | Ejigu T, etal/([26](#_ENREF_26)) | 2021 | Ethiopia | Amhara | diabetic patients | Crosssectional | interview & chart review  Ocular exam | Duration Diabetic illness | 2.91 | 1.068153 | 1.01 | 0.00995 | 8.35 | 2.122262 | 2.070065 | Not reported | 8.0 |
| 5 | Gelcho GN, et al./([30](#_ENREF_30)) | 2022 | Ethiopia | Oromia | diabetic patients | Cohort | Record review | Duration Diabetic illness | 2.86 | 1.050822 | 1.41 | 0.34359 | 5.31 | 1.669592 | 1.299482 | Not funded | 7.6 |

**Notes;** AOR; Adjusted odds ratio, CI; Confidence Interval, PGC- Poor glycemic control, SNNP: Southern Nations, Nationalities and Peoples’
