# Supplementary material for: Determinants of diabetic retinopathy in Ethiopia: A systematic review and meta-analysis
Source: PLoS One. 2023 Jun 8;18(6):e0286627. doi: 10.1371/journal.pone.0286627 (PMC10249865; doi:10.1371/journal.pone.0286627)
Supplement: Attachment — Submitted filename: PONE-D-22-31170_Sys_rev_DR.pdf [file pone.0286627.s004.pdf]

PLOS ONE

Determinants of Diabetic Retinopathy in Ethiopia; A systematic review and meta-analysis

--Manuscript Draft--

**Manuscript Number:**	PONE-D-22-31170
**Article Type:**	Research Article
**Full Title:**	Determinants of Diabetic Retinopathy in Ethiopia; A systematic review and meta-analysis
**Short Title:**	Determinants of Diabetic Retinopathy in Ethiopia
**Corresponding Author:**	Abere Woretaw Azagew<br>University of Gondar<br>ETHIOPIA
**Keywords:**	diabetes mellitus, diabetic retinopathy, determinants, risk factors, Ethiopia.
**Abstract:**	Introduction<br>Diabetic retinopathy (DR) is the primary retinal vascular complication, is the leading cause of visual impairment and blindness among diabetic patients. It affects the global population. In Ethiopia, DR is the most common public health problem among diabetic patients, but little is known about the determinant factors of DR. Therefore, we aimed to identify the risk factors for DR among diabetic patients in Ethiopia.<br>Methods: We have accessed previous studies through an electronic web-based search strategy using PubMed, Google (Scholar), the Web of Science, and the Cochrane Library with a combination of searching terms. The quality of each included article was assessed using the Newcastle Ottawa Assessment Scale. All statistical analyses were carried out using STATA version 14 software. The Odds ratios (ORs) of major risk factors were pooled using random-effects meta-analysis. Heterogeneity was assessed using Cochrane Q statistics and I-Square (I2). Furthermore, publication bias was detected based on the graphic asymmetry test of the funnel plot and/or Egger's test (p< 0.05).<br>Results: The search strategy retrieved 1182 articles. After the removal of duplicate articles, 249 articles remained. Following further screening, about 18 articles were assessed for eligibility, of which three articles were excluded because of reporting without the outcome of interest, poor quality, and not full text. Finally, fifteen studies were reviewed for the final analysis. Co-morbid hypertension (HTN) (AOR 2.04, 95%CI: 1.07, 3.89), poor glycemic control (AOR=4.36, 95%CI: 1.47, 12.90), and duration of diabetes illness (AOR =3.83, 95%CI: 1.17, 12.55) were found to be a confirmed associated factors of diabetic retinopathy.<br>Conclusion; In this study, co-morbid HTN, poor glycemic control and longer duration of diabetes illness were found to be the determinant factors of DR. Aggressive treatment of co-morbid HTN and blood glucose, and regular eye screening should be implemented to reduce the occurrence of DR among diabetic patients.
**Order of Authors:**	Abere Woretaw Azagew
	Yeabat Birhanu Yeabat Birhanu Yohannes
	Zerko Wako Beko
	Yohannes Mulu Ferede
	Chilot Kassa Mekonnen
**Opposed Reviewers:**	
**Additional Information:**	
**Question**	**Response**
**Financial Disclosure**<br><br>Enter a financial disclosure statement that	The author(s) received no specific funding for this work.

describes the sources of funding for the work included in this submission. Review the submission guidelines for detailed requirements. View published research articles from *PLOS ONE* for specific examples.<br><br>This statement is required for submission and **will appear in the published article** if the submission is accepted. Please make sure it is accurate.<br><br>**Unfunded studies**<br>Enter: *The author(s) received no specific funding for this work.*<br><br>**Funded studies**<br>Enter a statement with the following details:<br>• Initials of the authors who received each award<br>• Grant numbers awarded to each author<br>• The full name of each funder<br>• URL of each funder website<br>• Did the sponsors or funders play any role in the study design, data collection and analysis, decision to publish, or preparation of the manuscript?<br>• **NO** - Include this sentence at the end of your statement: *The funders had no role in study design, data collection and analysis, decision to publish, or preparation of the manuscript.*<br>• **YES** - Specify the role(s) played.<br><br>* typeset	
**Competing Interests**<br><br>Use the instructions below to enter a competing interest statement for this submission. On behalf of all authors, disclose any competing interests that could be perceived to bias this work—acknowledging all financial support and any other relevant financial or non-financial competing interests.<br><br>This statement is required for submission and **will appear in the published article** if the submission is accepted. Please make sure it is accurate and that any funding	The authors have declared that no competing interests exist.

Powered by Editorial Manager® and ProduXion Manager® from Aries Systems Corporation

sources listed in your Funding Information later in the submission form are also declared in your Financial Disclosure statement.<br><br>View published research articles from *PLOS ONE* for specific examples.<br><br>**NO authors have competing interests**<br><br>Enter: *The authors have declared that no competing interests exist.*<br><br>**Authors with competing interests**<br><br>Enter competing interest details beginning with this statement:<br><br>*I have read the journal's policy and the authors of this manuscript have the following competing interests: [insert competing interests here]*<br><br>* typeset	
**Ethics Statement**<br><br>Enter an ethics statement for this submission. This statement is required if the study involved:<br><br>• Human participants<br>• Human specimens or tissue<br>• Vertebrate animals or cephalopods<br>• Vertebrate embryos or tissues<br>• Field research<br><br>Write "N/A" if the submission does not require an ethics statement.<br><br>General guidance is provided below. Consult the submission guidelines for detailed instructions. **Make sure that all information entered here is included in the Methods section of the manuscript.**	N/A

**Format for specific study types**<br><br>**Human Subject Research (involving human participants and/or tissue)**<br>• Give the name of the institutional review board or ethics committee that approved the study<br>• Include the approval number and/or a statement indicating approval of this research<br>• Indicate the form of consent obtained (written/oral) or the reason that consent was not obtained (e.g. the data were analyzed anonymously)<br><br>**Animal Research (involving vertebrate animals, embryos or tissues)**<br>• Provide the name of the Institutional Animal Care and Use Committee (IACUC) or other relevant ethics board that reviewed the study protocol, and indicate whether they approved this research or granted a formal waiver of ethical approval<br>• Include an approval number if one was obtained<br>• If the study involved *non-human primates*, add *additional details* about animal welfare and steps taken to ameliorate suffering<br>• If anesthesia, euthanasia, or any kind of animal sacrifice is part of the study, include briefly which substances and/or methods were applied<br><br>**Field Research**<br><br>Include the following details if this study involves the collection of plant, animal, or other materials from a natural setting:<br>• Field permit number<br>• Name of the institution or relevant body that granted permission	
**Data Availability**<br><br>Authors are required to make all data underlying the findings described fully available, without restriction, and from the time of publication. PLOS allows rare exceptions to address legal and ethical concerns. See the PLOS Data Policy and FAQ for detailed information.	Yes - all data are fully available without restriction

A Data Availability Statement describing where the data can be found is required at submission. Your answers to this question constitute the Data Availability Statement and **will be published in the article**, if accepted.<br><br>**Important:** Stating 'data available on request from the author' is not sufficient. If your data are only available upon request, select 'No' for the first question and explain your exceptional situation in the text box.<br><br>Do the authors confirm that all data underlying the findings described in their manuscript are fully available without restriction?	
**Describe where the data may be found in full sentences. If you are copying our sample text, replace any instances of XXX with the appropriate details.**<br><br>• If the data are **held or will be held in a public repository**, include URLs, accession numbers or DOIs. If this information will only be available after acceptance, indicate this by ticking the box below. For example: *All XXX files are available from the XXX database (accession number(s) XXX, XXX.*).<br>• If the data are all contained **within the manuscript and/or Supporting Information files**, enter the following: *All relevant data are within the manuscript and its Supporting Information files.*<br>• If neither of these applies but you are able to provide **details of access elsewhere**, with or without limitations, please do so. For example:<br><br>*Data cannot be shared publicly because of [XXX]. Data are available from the XXX Institutional Data Access / Ethics Committee (contact via XXX) for researchers who meet the criteria for access to confidential data.*<br><br>*The data underlying the results presented in the study are available from (include the name of the third party*	All relevant data are within the manuscript and its Supporting Information files.

and contact information or URL).<br>• This text is appropriate if the data are owned by a third party and authors do not have permission to share the data.<br><br>* typeset	
Additional data availability information:	

Powered by Editorial Manager® and ProduXion Manager® from Aries Systems Corporation

Date: **November 11/ 2022**

To: **PLOS ONE**

Subject: **Submission of Manuscript**

Dear Editors,

We are delighted to submit a research entitled with“**Determinants of Diabetic Retinopathy in Ethiopia; A systematic review and meta-analysis”** for your possible evaluation.

The authors understand that the material presented in this paper has not been published and not submitted to any other journals. We confirm that this work has been approved by all authors.

The authors also understand that this manuscript is appropriate for **“PLOS ONE”** since the journal is a leading open-access journal, providing an innovative and influential venue for research and comment on the major challenges to human health worldwide.

I hope that the reviewing process finds the manuscript acceptable for publication in the journal.

Sincerely

Abere Woretaw Azagew (Corresponding author)

E mail: wabere@ymail.com

Manuscript
Click here to access/download;Manuscript;Manuscript.docx

Determinants of Diabetic Retinopathy in Ethiopia: A systematic review and meta-analysis

Abere Woretaw Azagew [1*], Yeabat Birhanu Yohannes [1], Zerko Wako Beko [1], Yohannes Mulu Ferede, Chilot Kassa Mekonnen[1]

[1]Department of Medical Nursing, School of Nursing, College of Medicine and Health Sciences, University of Gondar

Email address; wabere@ymail.com, birhanuyeneabat@gmail.com, zerkowako1992@gmail.com, yohannesmulu549@yahoo.com, chilotkassa.m@gmail.com,

Corresponding author: Abere Woretaw Azagew

Email: wabere@ymail.com

Abstract

Introduction

Diabetic retinopathy (DR) is the primary retinal vascular complication, and a ~~is the~~ leading cause of visual impairment and blindness among diabetic patients. It affects the global population. In Ethiopia, DR is the most common public health problem among diabetic patients, but little is known about the determinants ~~factors~~ of DR. Therefore, we aimed to identify the risk factors for DR among diabetic patients in Ethiopia.

Methods: We have accessed previous studies through an electronic web-based search strategy using PubMed, Google (Scholar), the Web of Science, and the Cochrane Library with a combination of search ~~searching~~ terms. The quality of each included article was assessed using the Newcastle Ottawa Assessment Scale. All statistical analyses were carried out using STATA version 14 software. The Odds ratios (ORs) of major risk factors were pooled using random-effects meta-analysis. Heterogeneity was assessed using Cochrane Q statistics and I-Square (I^2). Furthermore, publication bias was detected based on the graphic asymmetry test of the funnel plot and/or Egger's test ($p < 0.05$).

Results: The search strategy retrieved 1182 articles. After the removal of duplicate articles, 249 articles remained. Following further screening, about 18 articles were assessed for eligibility, of which three articles were excluded because of reporting without the outcome of interest, poor quality, and not full text. Finally, fifteen studies were reviewed for the final analysis. Co-morbid hypertension (HTN) (AOR 2.04, 95%CI: 1.07, 3.89), poor glycemic control (AOR=4.36, 95%CI: 1.47, 12.90), and duration of diabetes illness (AOR =3.83, 95%CI: 1.17, 12.55) were found to be a confirmed associated factors of diabetic retinopathy.

Conclusion; In this study, co-morbid HTN, poor glycemic control and longer duration of diabetes illness were found to be the determinant factors of DR. Aggressive treatment of co-morbid HTN and blood glucose, and regular eye screening should be implemented to reduce the occurrence of DR among diabetic patients.

Keywords: diabetes mellitus, diabetic retinopathy, determinants, risk factors, Ethiopia.

Introduction

Diabetic retinopathy (DR) is one of the most common microvascular complications of diabetes mellitus that affects the eye (retina) and is the leading cause of visual impairment or blindness among working age individuals worldwide (1-3). Almost all type 1 diabetic patients and >60% of type 2 diabetic patients have DR (4). Globally, the prevalence of DR was (22.27%) (5), and it was relatively highest in the Africa region (33.8%) (6). In Ethiopia, the prevalence reached (19.48%) (7).

The main cause of DR is diabetes; it occurs when the amount of blood glucose is too high. Diabetic retinopathy initially starts with the diagnosis of retinal capillaries and then progress to the larger blood vessels. The earlier stages of DR are characterized by the formation of microaneurysms, capillary leakage resulting in intraretinal hemorrhages, hard exudates, retinal edema, and capillary occlusion resulting in ischemia and wool spot formation, and it end up with loss of vision if left untreated. (8, 9).

A patient with diabetes is at risk of developing diabetic retinopathy. Several studies have looked into poor glycemic control (10), longer duration of diabetes treatment(10-15), HbA1c (15, 16), presence of HTN (11, 13, 15), high blood lipid (11, 12, 16), overweight/obesity (14), and smoking(11)are the most important risk factors for the development of DR. As diabetic

retinopathy worsens, the patient's vision becomes poorer, colors fade, the number of floaters increases, and the vision becomes blurry (17).

Intensive glycemic control reduces the incidence and progression of microvascular complications (18). So, it is possible to reduce the risk of developing diabetic retinopathy or help stop it from getting worse by keeping blood glucose, blood pressure, and cholesterol levels stable; also, adopting a healthy lifestyle by eating a healthy and balanced diet, losing weight, exercising regularly, stopping smoking, limiting alcohol intake, and adhering to medications are means of controlling the incidence of DR (19). Moreover, the effects of major risk factors for DR are still discrepant and inconclusive in Ethiopia, and need to be systematically evaluated in an evidence-based fashion. Based on the existing evidence, we attempted ~~tried~~ to retrieve the studies reported on DR to fill the gaps outlined above. Therefore, the aim of this study is to identify the major determinant factors of DR among diabetic patients in Ethiopia.

Methods

Data sources and search strategy

The procedure for this systematic review and meta-analysis was designed in accordance with the Preferred Reporting Items for Systematic Review and Meta-Analysis (PRISMA) flow chart (20). We searched on PubMed, Google, Google Scholar, Web of Science, and Cochrane Library databases for studies reporting on risk factors for diabetic retinopathy. Endnote (Version 7) reference management software was used to download, organize, review, and cite the articles Our comprehensive search strategies combined terms of diabetic retinopathy: epidemiology (incidence, prevalence, magnitude, morbidity, mortality), factors (determinants, risk factors, associated factors), and Ethiopia using both controlled vocabularies (eg, Medical Subject

Heading terms) and free text words. The articles published from 2015 to 2022 were included in this study. Boolean operators like “AND” and “OR” were used to combine search terms.

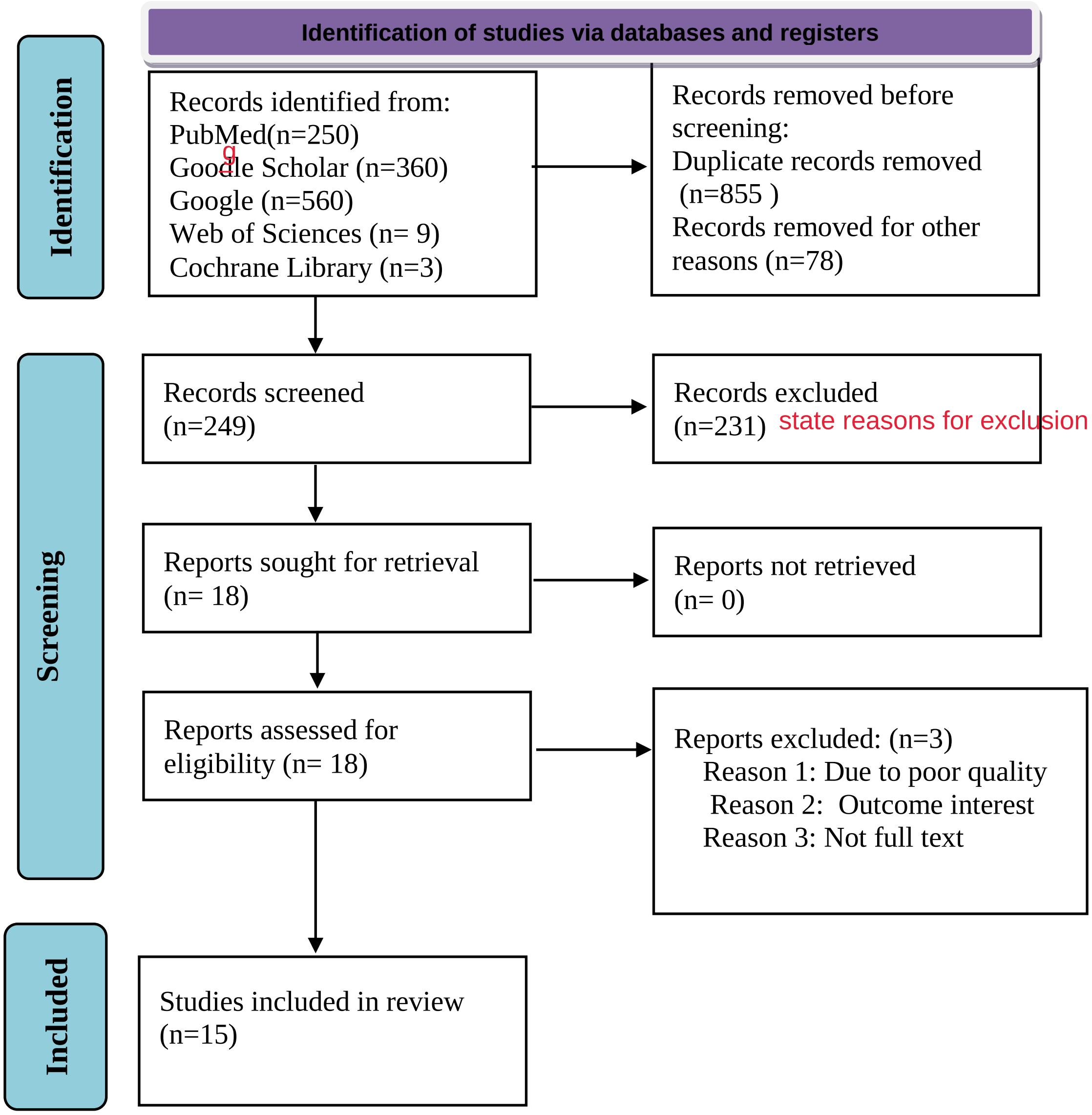

Fig 1: PRISMA flow chart for flow of information through the phases of systematic review

Inclusion and exclusion criteria

Those published or unpublished articles on risk factors for diabetic retinopathy conducted in Ethiopia were included in the study. Articles that reported the determinants, risk factors, and prevalence of diabetic retinopathy were included in the study. Information from conference papers, articles without full texts, and qualitative studies were excluded from the study.

Data extraction

Two reviewers (CKM and YBY) screened the titles and abstracts of identified studies and assessed the full-texts of potentially eligible studies. Any controversy was resolved by consensus. An effort was made to communicate with the authors whenever further information was needed. The data were extracted independently by these reviewers using data extraction format. Using Microsoft Excel, data on the author(s), study year or year of publication, study design, sample size, and prevalence or determinant factors of diabetic retinopathy were extracted from eligible articles. The whole manuscript was critically reviewed by all authors.

Quality assessment

Articles were assessed for quality score using the New Castle Ottawa Scale adapted for cross-sectional studies' quality assessment tool; a score of ≥ 7 out of 10 was considered a high-quality score (21). Two authors (CKM and HMA) assessed the quality of each paper. The reviewers compared the quality of the appraisal scores and resolved inconsistencies prior to calculating the final appraisal score. All the included studies had high quality scores. The PRISMA checklist 2020 (22) was used to report the results of this study

Data analysis

The data were analyzed using STATA version 11. The summary effect estimates of the prevalence were calculated using meta-analysis in a random effect model (23). Cochran's Q statistic and I-squared (I^2) were used to evaluate the presence of heterogeneity. The I^2 square test statistic results of 25%, 50%, and 75% were declared as low, moderate, and high heterogeneity (24), respectively. The Odds ratios (ORs) of major risk factors were pooled using random-effects meta-analysis. A random effect model was used to minimize the variation across the study. Subgroup analyses by profession were done to estimate the variations in the knowledge status of healthcare workers. Furthermore, sensitivity analysis was used to see the discrepancy among studies. The publication bias was detected based on the graphic asymmetry test of the funnel plot and/or Egger's test ($p< 0.05$) (25).

Results

Study selection and characteristics

The search strategy retrieved 1182 articles. After the removal of duplicate articles, 249 articles remained. Following additional screening, eighteen articles were evaluated for eligibility, with three being excluded due to being incomplete, of poor quality, or not in full text. Finally, fifteen studies were reviewed for co-morbid HTN (1, 2, 26-38) (**Table 1**), six studies for poor glycemic control (1, 2, 27, 28, 31, 35) (**Table 2**), and five studies for duration of diabetic illness(1, 2, 28, 30, 34) (**Table 3**). Of the total studies; five were conducted in the Amhara region (1, 26, 30, 33, 37), five in Addis Ababa (27, 29, 32, 35, 38), three in the Oromia region (28, 34, 36), and two in South Nations and Nationalities people (SNNP) of Ethiopia (2, 31).

All the studies were published between the years 2015 and 2022. Regarding the study design, eight studies ~~are~~ were cross sectional (1, 2, 26, 29, 30, 35, 37, 38), five studies ~~are~~ were cohort (31-34, 36), and two studies ~~are~~ were case control (27, 28).

Table 1: Study characteristics by co-morbid HTN among diabetic patients in Ethiopia

Author/year	Study design	Region of the study	Factors	AOR	95% CI
Tilahun M, etal/2020(1)	Cross sectional	Amhara	Co-HTN	3.39	1.64-7.02
Alemayehu HB, etal./2022(2)	Cross sectional	SNNP	Co-HTN	1.43	0.72-2.86
Mersha GA, etal./2021(26)	Cross sectional	Amhara	Co-HTN	1.67	0.66-4.20
Seid K, etal./2021(27)	case control	Addis Ababa	Co-HTN	12.3	6.95-21.8
Garoma D etal./2020(28)	Case control	Oromia	Co-HTN	3.38	1.29-9.05
Shibru T, etal./2018(29)	Cross sectional	Addis Ababa	Co-HTN	2.556	1.014-6.447
Ejigu T, etal/2021(30)	Cross sectional	Amhara	Co-HTN	2.65	1.02-6.87
Chisha Y, etal./2017(31)	Cohort	SNNP	Co-HTN	4.1	1.76-9.44
Azeze TK, etal./2018(32)	Cohort	Addis Ababa	Co-HTN	1.51	0.48-4.74
Takele MB, etal./2022(33)	Cohort	Amhara	Co-HTN	1.68	1.14-2.50
Gelcho GN, etal./2022(34)	Cohort	Oromia	Co-HTN	2.32	1.12-4.39
Aberra T, etal./2022(35)	Cross sectional	Addis Ababa	Co-HTN	1.37	0.865-2.169
Debele GR,etal./2021(36)	Cohort	Oromia	Co-HTN	0.54	0.35-0.82
Alemu S, etal./2015(37)	Cross sectional	Amhara	Co-HTN	5.2	2.5-10.20
Abera F, etal./2021(38)	Cross sectional	Addis Ababa	Co-HTN	8.63	2.51-29.75

Notes; AOR; Adjusted odds ratio, CI; Confidence Interval, Co-HTN: Co-morbid Hypertension, SNNP: Southern Nations, Nationalities and Peoples'

Table 2: Study characteristics by poor glycemic control status among diabetic patients in Ethiopia.

Author/year	Study design	Region of the study	Factors	AOR	95% CI
Tilahun M, etal/2020(1)	Cross sectional	Amhara	PGC	4.58	1.86-11.31
Alemayehu HB, etal./2022(2)	Cross sectional	SNNP	PGC	4.34	2.26-8.34
Mersha GA, etal./2021(26)	Cross sectional	Amhara	PGC	3.2	1.5-6.7

Seid K, etal./2021(27)	case control	Addis Ababa	PGC	10.7	6.17-18.58
Garoma D etal./2020(28)	Case control	Oromia	PGC	9.08	3.7-22.29
Aberra T, etal./2022(35)	Cross sectional	Addis Ababa	PGC	1.23	0.667-2.276

Notes; AOR; Adjusted odds ratio, CI; Confidence Interval, PGC: Poor glycemic control, SNNP: Southern Nations, Nationalities and Peoples'

Table 3: Study characteristics by Duration of Diabetic illness among diabetic patients in Ethiopia

Author/year	Study design	Region of the study	Factors	AOR	95% CI
Tilahun M, etal/2020(1)	Cross sectional	Amhara	Duration Diabetic illness	3.91	1.86-8.23
Alemayehu HB, etal./2022(2)	Cross sectional	SNNP	Duration Diabetic illness	4.78	2.11-10.83
Garoma D etal./2020(28)	Case control	Oromia	Duration Diabetic illness	4.38	2.65-7.22
Ejigu T, etal/2021(30)	Cross sectional	Amhara	Duration Diabetic illness	2.91	1.01-8.35
Gelcho GN, etal./2022(34)	Cohort	Oromia	Duration Diabetic illness	2.86	1.41-5.31

Notes; AOR; Adjusted odds ratio, CI; Confidence Interval, PGC- Poor glycemic control, SNNP: Southern Nations, Nationalities and Peoples'

Determinants of diabetic retinopathy

In this systematic review and meta-analysis, co-morbid HTN is found to be the determinant factor for diabetic retinopathy. Diabetic patients who have co-morbid HTN are 2.04 times more likely to have diabetic retinopathy compared to those diabetic patients with no co-morbid HTN (AOR 2.04, 95%CI: 1.07, 3.89) (**Fig 2**).

.Heterogeneity and publication bias of included studies

The overall heterogeneity test (**I^2**) on the effect of co-morbid HTN was 0.0% with a $p < 0.946$, using a random effect model to adjust observed variability. This indicates there is no variability across the studies.

Regarding the publication bias, the graphic asymmetry test of the funnel plot shows a symmetrical distribution (**Fig** 3), and Egger's test p value= 0.181, indicating that there is no publication bias.

Poor glycemic control

In this study, poor glycemic control ~~is~~ was found to be the determinant factor for diabetic retinopathy. Diabetic patients with poor glycemic control status ~~are~~ were 4.36 times more likely to have diabetic retinopathy compared to those diabetic patients with good glycemic control status (AOR=4.36, 95%CI: 1.47, 12.90) (**Fig.** 4).

Heterogeneity and publication bias of included studies

The heterogeneity test (**I^2**) on the effect of poor glycemic control was 0.0% with a p value< 0.847, using a random effect model to adjust observed variability. This indicates there is no variability across the studies. The publication bias was assessed using the graphic asymmetry test of the funnel plot, shows a symmetrical distribution (**Fig 5**), and Egger's test p value = 0.851 means that there ~~is~~ was no publication bias.

Duration of diabetic illness

Longer duration of diabetic illness ~~is~~ was found to be the risk factor for the development of ~~D~~d iabetic retinopathy. Patients who ~~have~~ had longer duration of diabetic illness ~~are~~ were nearly four times more likely to have diabetic retinopathy compared to those diabetic patients with shorter duration of diabetic illness (AOR =3.83, 95%CI: 1.17, 12.55, I^2=0.0% and P value = 0.999) (**Fig** 6).

Heterogeneity and publication bias of included studies

The overall heterogeneity test (**I**^{2}) on the effect of co-morbid hypertension was 0.0% with a p value< 0.999, using a random effect model to adjust observed variability. This indicates there ~~is~~ was no variability across the studies. Concerning the publication bias, the graphic asymmetry test of the funnel plot shows a symmetrical distribution (**Fig** 7) and Egger's test p value= 0.624, indicating that there is no publication bias.

Discussion

Diabetic patients experience different complications along the continuum of their lives, of which diabetic retinopathy is the most common ~~one~~. We tried to investigate the determinant factors of diabetic retinopathy. In this systematic review and meta-analysis, co-morbid HTN, poor glycemic control status, and longer duration of diabetic illness ~~are~~ found to be the determinate factors of diabetic retinopathy.

The pooled effect of this study depicts that diabetic patients who have co-morbid HTN ~~are~~ 2.04 times more likely to have diabetic retinopathy compared to those diabetic patients with no co-morbid HTN. The finding of this study is supported by large scale study in China (39), and (Chinese, Malay and India) (40). This is the fact that HTN has an impact on retinal blood vessels. It damages the retinal vascular structures. It is an indicator of target organ damage (41). An increase in blood pressure is transferred directly to the vessels, which initially constrict. However, a further increase in BP overcomes this compensatory tone, and damage to the muscle layer and endothelium ensues (42). This results in retinal edema, cotton wool spots, hemorrhage, and disc edema (43). Evidence showed that, a population based approach to tight blood pressure control in the diabetic population may reduce the incidence of sight threatening retinopathy with a favorable impact on the lives of diabetic patients. A decrease in every 10 mmHg of blood

pressure leads to a reduction in 35% of retinopathy, a 35% need for retinal laser, and 50% blindness (44).

Similarly, poor glycemic control status is the determinant factor for diabetic retinopathy. Diabetic patients who have poor glycemic control status are nearly 4.4 times more likely to have diabetic retinopathy compared to those diabetic patients with good glycemic control status. The finding of this study is supported by the study conducted in China (39, 45). The possible reason is that too much blood glucose in the blood may block the tiny blood vessels that nourish the retina. As a result, the eye attempts to grow new blood vessels, but these new blood vessels don't develop properly and can leak easily, which leads to vascular edema (46). Glucose toxicity is the key initial trigger for diabetic retinopathy. The endothelial cells malfunction owing to chronic exposure to high levels of glucose and other factors. The resulting lesions include thickened capillary basement membranes, defects in the blood-retinal barrier, and pericyte loss (47).

Furthermore, having diabetes for a longer period of time is a risk factor for diabetic retinopathy. Patients who have a longer duration of diabetic illness are nearly four times more likely to have diabetic retinopathy compared to those diabetic patients with a short duration of diabetic illness. This is supported by the study conducted in China (39, 48). Patients with diabetes develop retinopathy within the early stages of disease, but this does not affect the sight, unless the patient is treated, it progresses and eventually affects the sight (49).

The authors try to investigate the risks of diabetic retinopathy, and the evidence generated is important for policy makers and clinicians to act upon possible interventions to tackle the occurrence of DR. This study did not separate diabetic types, so further study will be needed specifically for each type of diabetic patient separately. Additionally, the prevalence of DR is not

included in this study. Lifestyle intervention is remarkably effective in the primary prevention of diabetes and HTN. The initial approach to management of both diabetes and HTN must emphasize on weight control, regular physical activity, and dietary modification (50). Healthcare workers should give due attention to those patients who have co-morbid HTN, poor glycemic control, and a longer duration of diabetic illness. Therefore, aggressive control of blood glucose and blood pressure, and regular eye screening are needed to prevent DR among diabetic patients.

Conclusion

In this systematic and meta-analysis study, co-morbid HTN, poor glycemic control, and longer duration of diabetes illness were found to be the determinant factors of DR. Therefore, aggressive treatment of co-morbid HTN and blood glucose, and regular eye screening should be implemented to reduce the occurrence of DR among diabetic patients.

Acknowledgements

We would like to acknowledge the team members for their invaluable contribution from the conception to the final approval for submission to publication.

Abbreviations

AOR-Adjusted Odds Ratio, CI-Confidence Interval, DR-Diabetic retinopathy, HbA1c-Glycated hemoglobin, HTN-Hypertension, OR-Odds Ratio, PRISMA-Preferred Reporting Item of Systematic Review and Meta-Analysis, SNNP-Southern Nations Nationalities and People

References

1. Tilahun M, Gobena T, Dereje D, Welde M, Yideg G. Prevalence of Diabetic retinopathy and its associated factors among diabetic patients at Debre Markos referral hospital, Northwest Ethiopia, 2019: hospital-based cross-sectional study. Diabetes, Metabolic Syndrome and Obesity: Targets and Therapy. 2020;13:2179.
2. Alemayehu HB, Tegegn MT, Tilahun MM. Prevalence and associated factors of visual impairment among adult diabetic patients visiting Adare General Hospital, Hawassa, South Ethiopia, 2022. Plos one. 2022;17(10):e0276194.
3. Hammer SS, Beli E, Kady N, Wang Q, Wood K, Lydic TA, et al. The Mechanism of Diabetic Retinopathy Pathogenesis Unifying Key Lipid Regulators, Sirtuin 1 and Liver X Receptor. EBioMedicine. 2017;22:181-90.
4. Fong DS, Aiello L, Gardner TW, King GL, Blankenship G, Cavallerano JD, et al. Retinopathy in diabetes. Diabetes care. 2004;27(suppl_1):s84-s7.
5. Teo ZL, Tham YC, Yu M, Chee ML, Rim TH, Cheung N, et al. Global Prevalence of Diabetic Retinopathy and Projection of Burden through 2045: Systematic Review and Meta-analysis. Ophthalmology. 2021;128(11):1580-91.
6. Thomas R, Halim S, Gurudas S, Sivaprasad S, Owens D. IDF Diabetes Atlas: A review of studies utilising retinal photography on the global prevalence of diabetes related retinopathy between 2015 and 2018. Diabetes research and clinical practice. 2019;157:107840.
7. Fite RO, Lake EA, Hanfore LK. Diabetic retinopathy in Ethiopia: a systematic review and meta-analysis. Diabetes & Metabolic Syndrome: Clinical Research & Reviews. 2019;13(3):1885-91.
8. Diabetic Retinopathy.The American Society of Retina Specialists 2022. Accessed date November 9/20222.
9. Natalia L. Pathophysiology of Diabetic Retinopathy. In: Giuseppe Lo G, editor. Diabetic Eye Disease. Rijeka: IntechOpen; 2022. p. Ch. 1.
10. Lima VC, Cavalieri GC, Lima MC, Nazario NO, Lima GC. Risk factors for diabetic retinopathy: a case–control study. International Journal of Retina and Vitreous. 2016;2(1):21.
11. Cai X-l, Wang F, Ji L-n. Risk factors of diabetic retinopathy in type 2 diabetic patients. Chinese medical journal. 2006;119(10):822-6.
12. Abougalambou SSI, Abougalambou AS. Risk factors associated with diabetic retinopathy among type 2 diabetes patients at teaching hospital in Malaysia. Diabetes & Metabolic Syndrome: Clinical Research & Reviews. 2015;9(2):98-103.
13. Shrestha MK, Paudyal G, Wagle RR, Gurung R, Ruit S, Onta SR. Prevalence of and factors associated with diabetic retinopathy among diabetics in Nepal: a hospital based study. Nepal Med Coll J. 2007;9(4):225-9.
14. Sahiledengle B, Assefa T, Negash W, Tahir A, Regasa T, Tekalegn Y, et al. Prevalence and Factors Associated with Diabetic Retinopathy among Adult Diabetes Patients in Southeast Ethiopia: A Hospital-Based Cross-Sectional Study. Clinical Ophthalmology. 2022:3527-45.
15. Tapp RJ, Shaw JE, Harper CA, De Courten MP, Balkau B, McCarty DJ, et al. The prevalence of and factors associated with diabetic retinopathy in the Australian population. Diabetes care. 2003;26(6):1731-7.
16. Tseng ST, Chou ST, Low BH, Su FL. Risk factors associated with diabetic retinopathy onset and progression in diabetes patients: a Taiwanese cohort study. International journal of clinical and experimental medicine. 2015;8(11):21507-15.
17. American Academy of Ophthalmology 2022. Diabetic Retinopathy: Causes, Symptoms, Treatment. Accessed date 9/2022. https://www.aao.org/eye-health/diseases/what-is-diabetic-retinopathy.

18. King P, Peacock I, Donnelly R. The UK prospective diabetes study (UKPDS): clinical and therapeutic implications for type 2 diabetes. British journal of clinical pharmacology. 1999;48(5):643.

19. Prevention. Diabetic retinopathy. Accsessed November 9/2022.https://www.nhs.uk/conditions/diabetic-retinopathy/prevention.

20. Page MJ, McKenzie JE, Bossuyt PM, Boutron I, Hoffmann TC, Mulrow CD, et al. The PRISMA 2020 statement: an updated guideline for reporting systematic reviews. Systematic reviews. 2021;10(1):1-11.

21. McPheeters ML, Kripalani S, Peterson NB, Idowu RT, Jerome RN, Potter SA, et al. Quality improvement interventions to address health disparities: closing the quality gap-revisiting the state of the science. Database of Abstracts of Reviews of Effects (DARE): Quality-assessed Reviews [Internet]. 2012.

22. Yepes-Nuñez J, Urrutia G, Romero-Garcia M, Alonso-Fernandez S. The PRISMA 2020 statement: an updated guideline for reporting systematic reviews. Revista Espanola de Cardiologia (English ed). 2021;74(9):790-9.

23. Borenstein M, Hedges LV, Higgins JP, Rothstein HR. A basic introduction to fixed-effect and random-effects models for meta-analysis. Research synthesis methods. 2010;1(2):97-111.

24. Rücker G, Schwarzer G, Carpenter JR, Schumacher M. Undue reliance on I 2 in assessing heterogeneity may mislead. BMC medical research methodology. 2008;8(1):1-9.

25. Begg CB, Mazumdar M. Operating characteristics of a rank correlation test for publication bias. Biometrics. 1994:1088-101.

26. Getasew Alemu Mersha YAA, Mikias Mered Tilahun, TarkegnCheklie Zeleke, Asamere Tsegaw Woredekal. Prevalence of Diabetic Retinopathy and Associated Risk Factors among Adult Diabetes Attending at Debre Tabor General Hospital, Northwest Ethiopia. Journal of Diabetes & Metabolism. May 25, 2021;14(4).

27. Seid K, Tesfaye T, Belay A, Mohammed H. Determinants of diabetic retinopathy in Tikur Anbessa Hospital, Ethiopia: a case–control study. Clinical Diabetes and Endocrinology. 2021;7(1):1-9.

28. Garoma D, Merga H, Hiko D. Determinants of diabetic retinopathy in Southwest Ethiopia: a facility-based case-control study. BMC Public Health. 2020;20(1):1-9.

29. Tsion Shiberu FA, & Abdissa Boka. Prevalence and factors associated with diabetes retinopathy among type 2 diabetes patients at Tikur Anbesa specialized hospital Addis Ababa, Ethiopia, 2018. 2018.

30. Ejigu T, Tsegaw A. Prevalence of diabetic retinopathy and risk factors among diabetic patients at University of Gondar Tertiary Eye Care and Training Center, North-West Ethiopia. Middle East African Journal of Ophthalmology. 2021;28(2):71.

31. Chisha Y, Terefe W, Assefa H. Incidence and factors associated with diabetic retinopathy among diabetic patients at arbaminch general hospital, gamo gofa Zone (longitudinal follow up data analysis). Journal of Diabetology. 2017;8(1):1.

32. Azeze TK, Sisay MM, Zeleke EG. Incidence of diabetes retinopathy and determinants of time to diabetes retinopathy among diabetes patients at Tikur Anbessa Hospital, Ethiopia: a retrospective follow up study. BMC research notes. 2018;11(1):1-6.

33. Takele MB, Boneya DJ, Alemu HA, Tsegaye TB, Birhanu MY, Alemu S, et al. Retinopathy among Adult Diabetics and Its Predictors in Northwest Ethiopia. Journal of Diabetes Research. 2022;2022.

34. Gelcho GN, Gari FS. Time to Diabetic Retinopathy and Its Risk Factors among Diabetes Mellitus Patients in Jimma University Medical Center, Jimma, Southwest Ethiopia. Ethiopian Journal of Health Sciences. 2022;32(5):937-46.

35. Aberra T, Feleke Y, Tarekegn G, Bikila D, Melesse M. Prevalence and associated factors of diabetic nephropathy at Tikur Anbessa Comprehensive Specialized University Hospital, Addis Ababa, Ethiopia. African Journal of Nephrology. 2022;25(1):35-45.

36. Debele GR, Kanfe SG, Weldesenbet AB, Ayana GM, Jifar WW, Raru TB. Incidence of diabetic retinopathy and its predictors among newly diagnosed type 1 and type 2 diabetic patients: a

retrospective follow-up study at tertiary health-care setting of Ethiopia. Diabetes, Metabolic Syndrome and Obesity: Targets and Therapy. 2021;14:1305.
37. Alemu S, Dessie A, Tsegaw A, Patterson CC, Parry EH, Phillips DI, et al. Retinopathy in type 1 diabetes mellitus: Major differences between rural and urban dwellers in northwest Ethiopia. Diabetes research and clinical practice. 2015;109(1):191-8.
38. FEKADU ABERA YT, & AKLIL HAILU. PREVALENCE OF DIABETIC RETINOPATHY AND ITS ASSOCIATED FACTORS AMONG TYPE TWO DIABETES MELLITUS PATIENTS IN HIWOT FANA SPECIALIZED UNIVERSITY HOSPITAL, HARAR, EASTERN ETHIOPIA, 2021. 2021.
39. Zhang G, Chen H, Chen W, Zhang M. Prevalence and risk factors for diabetic retinopathy in China: a multi-hospital-based cross-sectional study. British Journal of Ophthalmology. 2017;101(12):1591-5.
40. Liu L, Quang ND, Banu R, Kumar H, Tham Y-C, Cheng C-Y, et al. Hypertension, blood pressure control and diabetic retinopathy in a large population-based study. PLoS One. 2020;15(3):e0229665.
41. Grosso A, Veglio F, Porta M, Grignolo F, Wong T. Hypertensive retinopathy revisited: some answers, more questions. British Journal of Ophthalmology. 2005;89(12):1646-54.
42. Wong TY, Mitchell P. Hypertensive retinopathy. New England Journal of Medicine. 2004;351(22):2310-7.
43. Walsh JB. Hypertensive retinopathy: description, classification, and prognosis. Ophthalmology. 1982;89(10):1127-31.
44. Klein R, Knudtson MD, Lee KE, Gangnon R, Klein BE. The Wisconsin Epidemiologic Study of Diabetic Retinopathy XXII: the twenty-five-year progression of retinopathy in persons with type 1 diabetes. Ophthalmology. 2008;115(11):1859-68.
45. Song P, Yu J, Chan KY, Theodoratou E, Rudan I. Prevalence, risk factors and burden of diabetic retinopathy in China: a systematic review and meta-analysis. Journal of global health. 2018;8(1).
46. Shin ES, Sorenson CM, Sheibani N. Diabetes and retinal vascular dysfunction. Journal of ophthalmic & vision research. 2014;9(3):362.
47. Gillow J, Gibson J, Dodson P. Hypertension and diabetic retinopathy—what's the story? British journal of ophthalmology. 1999;83(9):1083-7.
48. Zoungas S, Woodward M, Li Q, Cooper ME, Hamet P, Harrap S, et al. Impact of age, age at diagnosis and duration of diabetes on the risk of macrovascular and microvascular complications and death in type 2 diabetes. Diabetologia. 2014;57(12):2465-74.
49. Klein R, Klein BE. Vision disorders in diabetes. Diabetes in America. 1995;1:293.
50. Long AN, Dagogo-Jack S. Comorbidities of diabetes and hypertension: mechanisms and approach to target organ protection. The journal of clinical hypertension. 2011;13(4):244-51.

Supporting Information – PRISMA checklist

Figure Click here to access/download;Figure;Fig 2 DR.tif

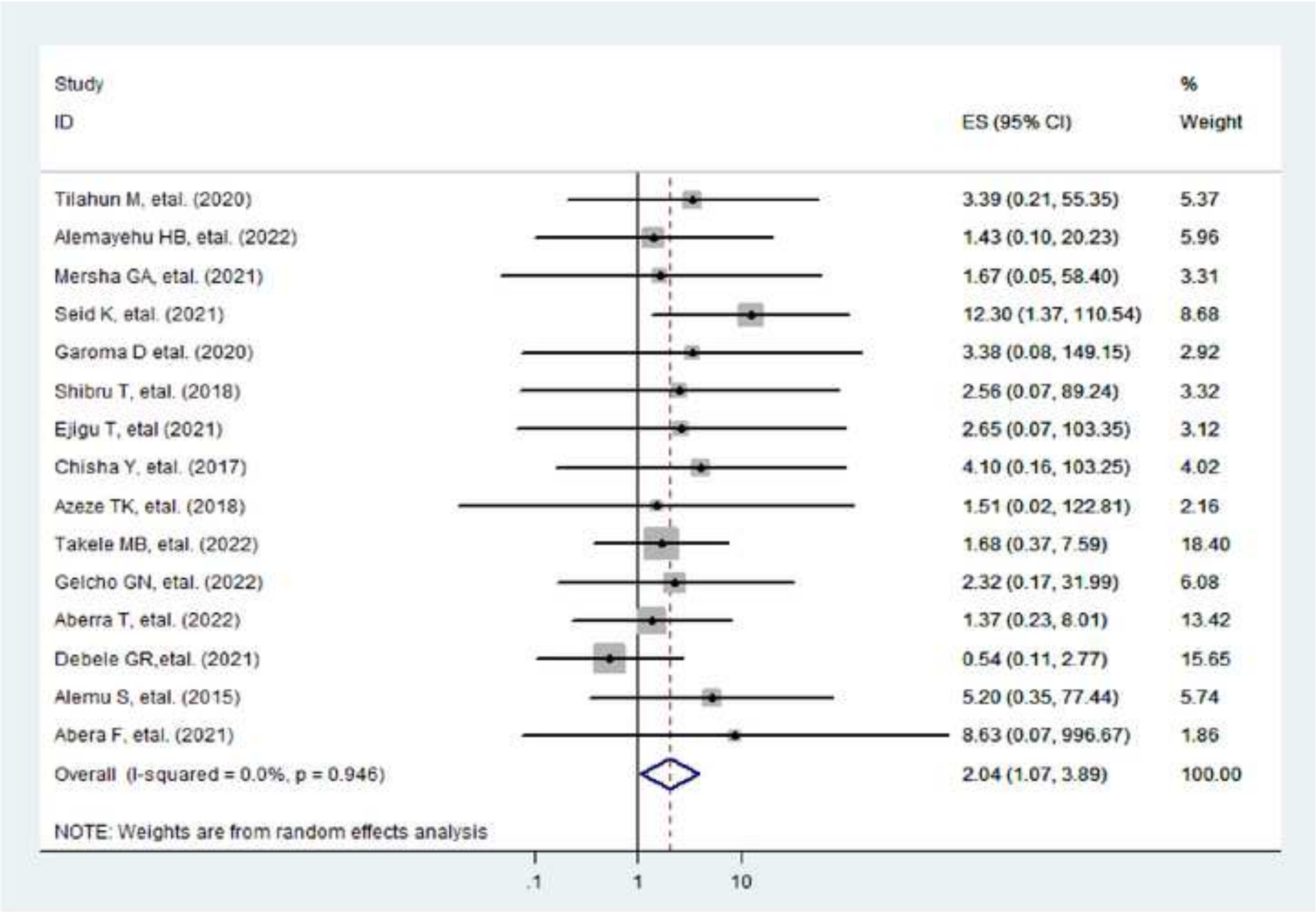

Fig 2. Forest plot shows the pooled effects of co-morbid hypertension on diabetic retinopathy.

Click here to access/download;Figure;Fig 3 DR.tif

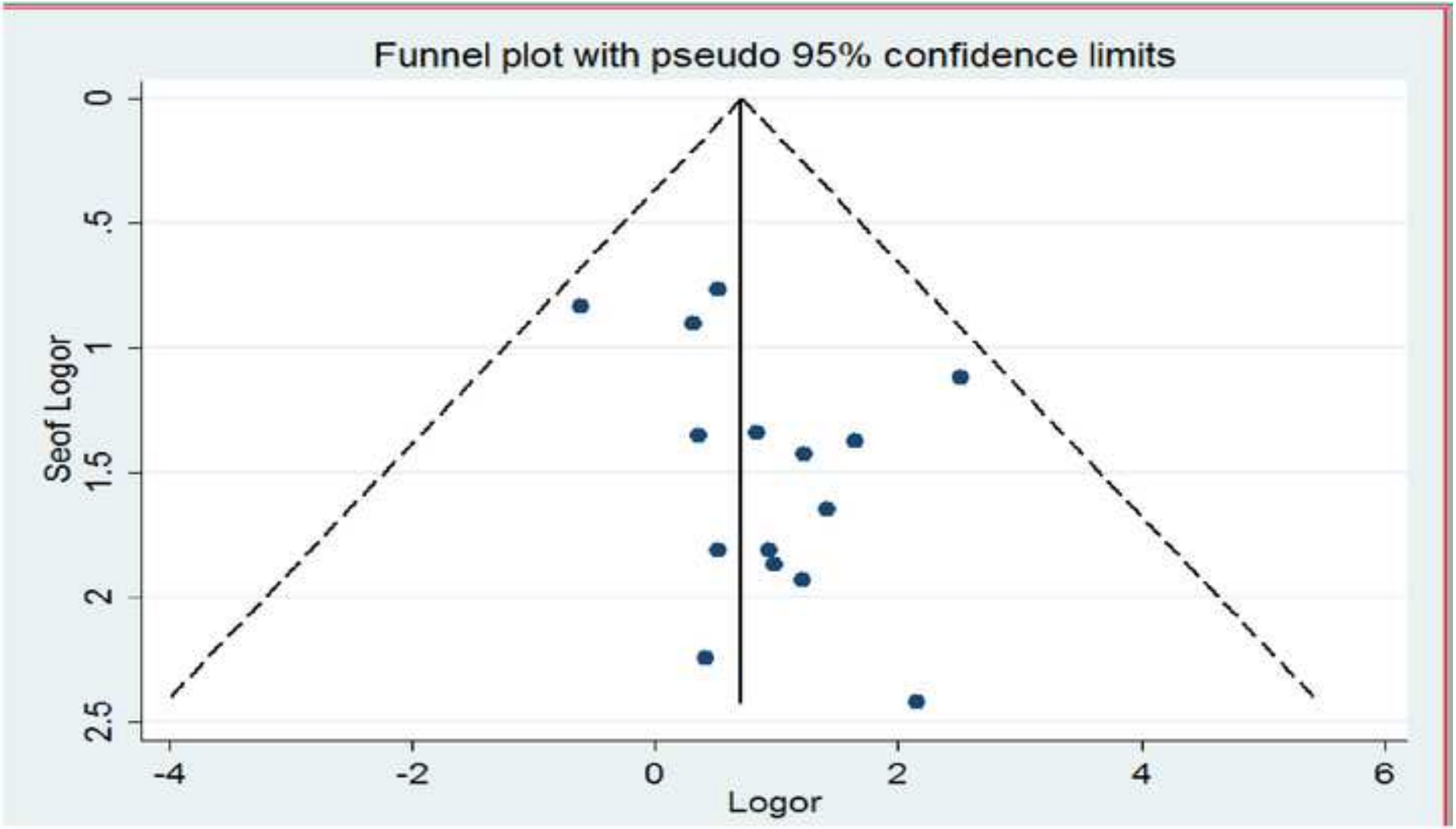

Fig 3: Funnel plot to assess the heterogeneity of the included studies

Figure

Click here to access/download;Figure;Fig 4 DR.tif

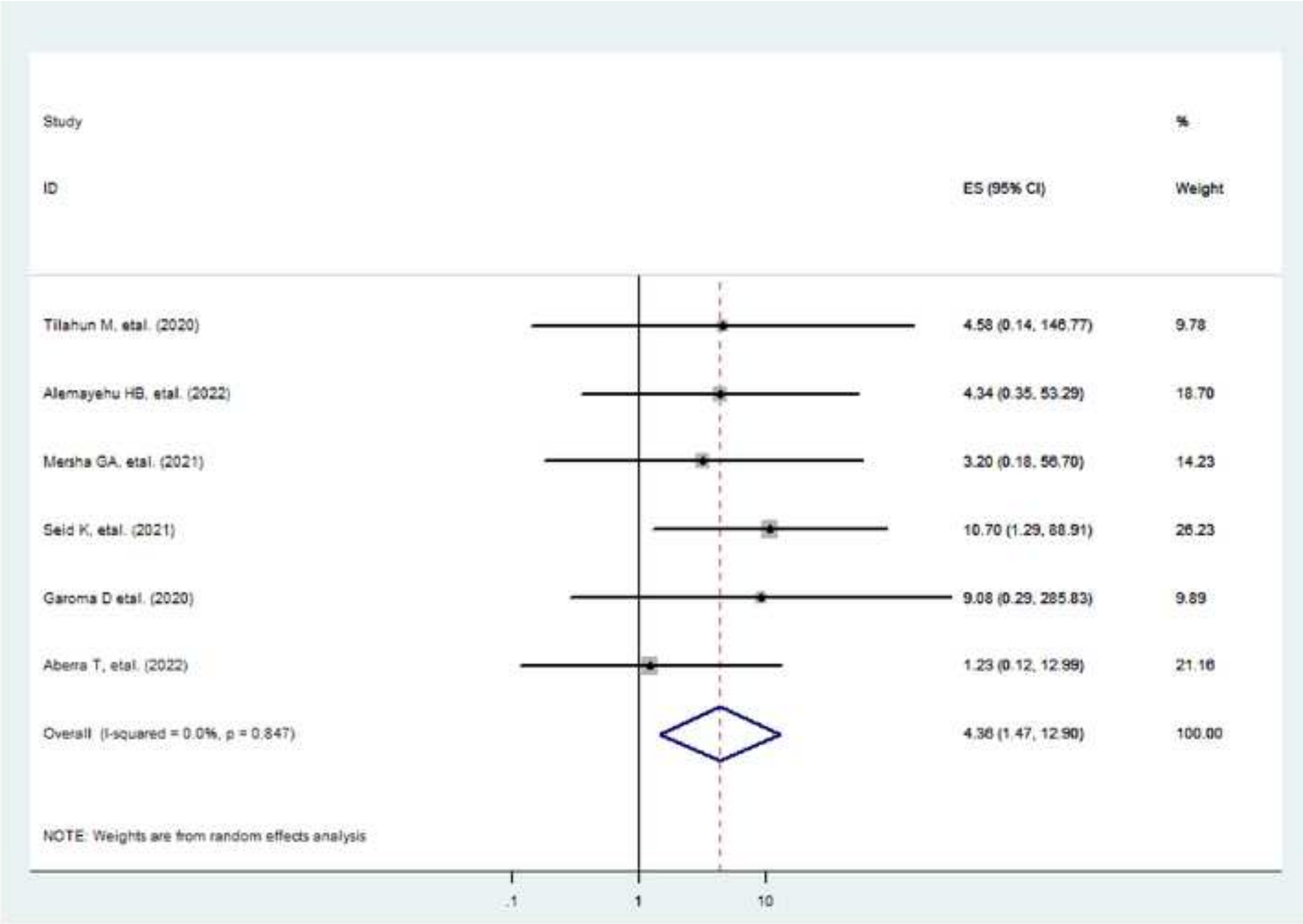

Fig 4. Forest plot shows the pooled effect of poor glycemic control on diabetic retinopathy.

 Click here to access/download;Figure;Fig 5 DR.tif

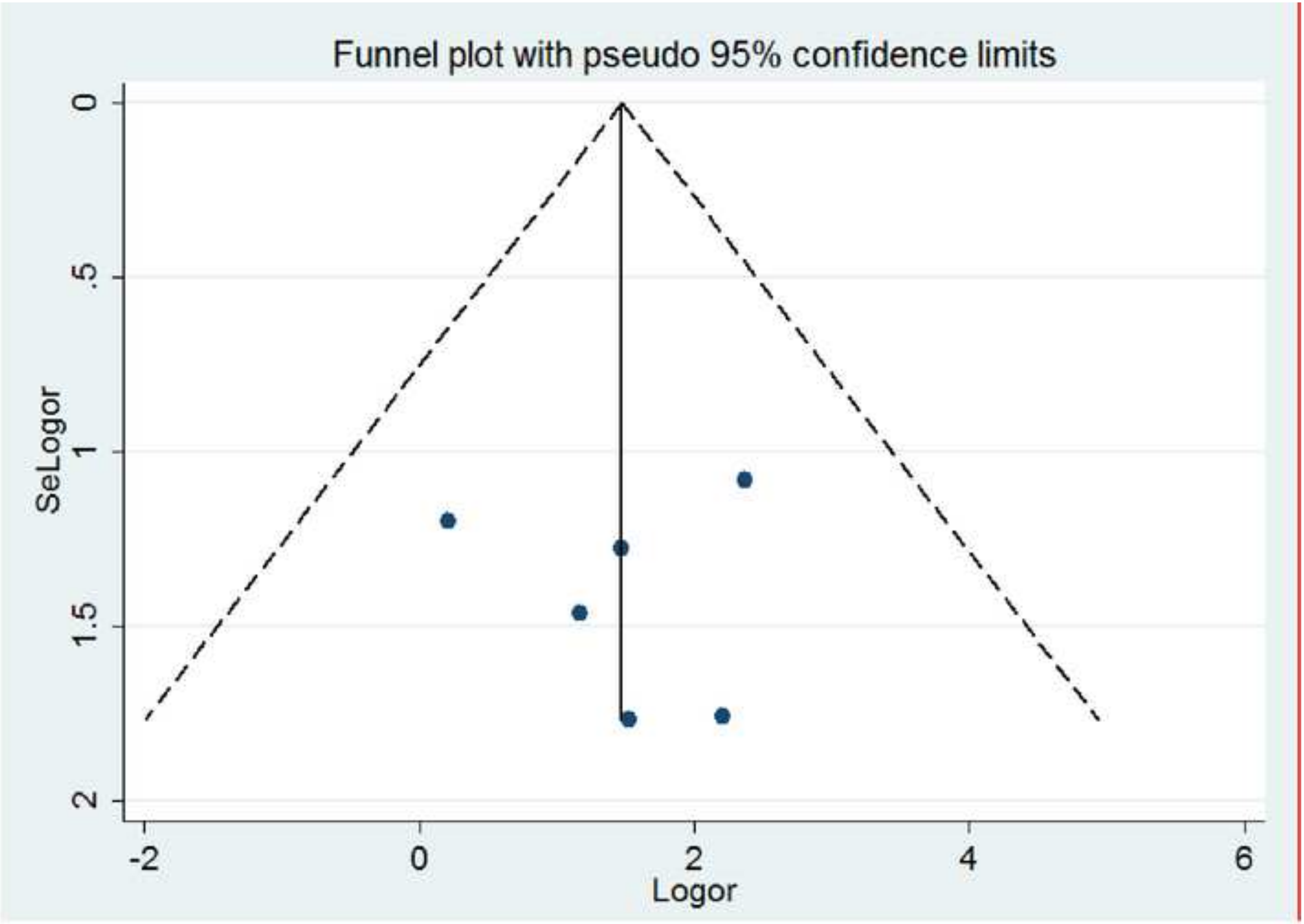

Fig 5: Funnel plot to assess the heterogeneity of the included studies

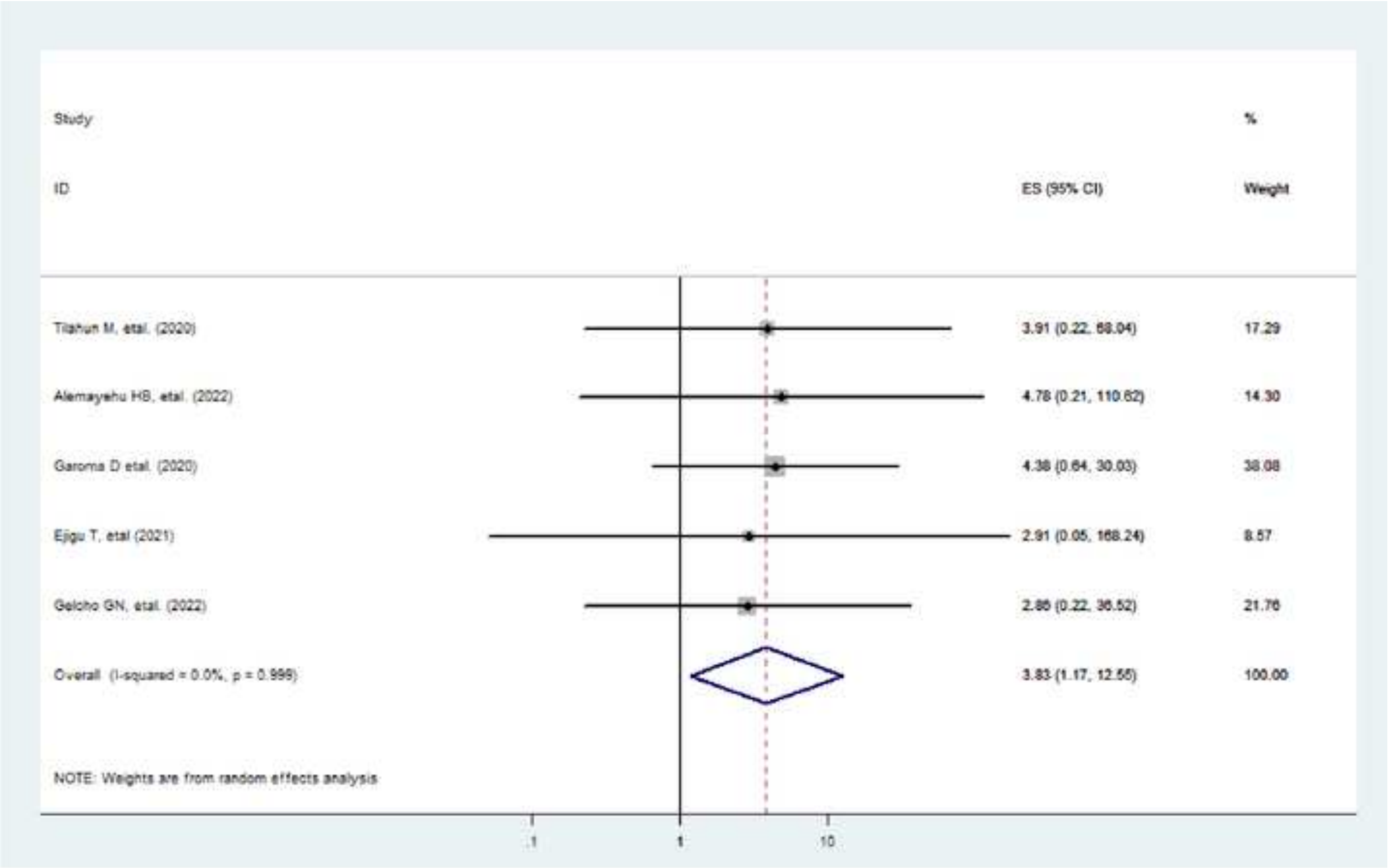

Fig 6: Forest plot shows the pooled effect of the duration diabetic illness on diabetic retinopathy

Click here to access/download;Figure;Fig 7 DR.tif

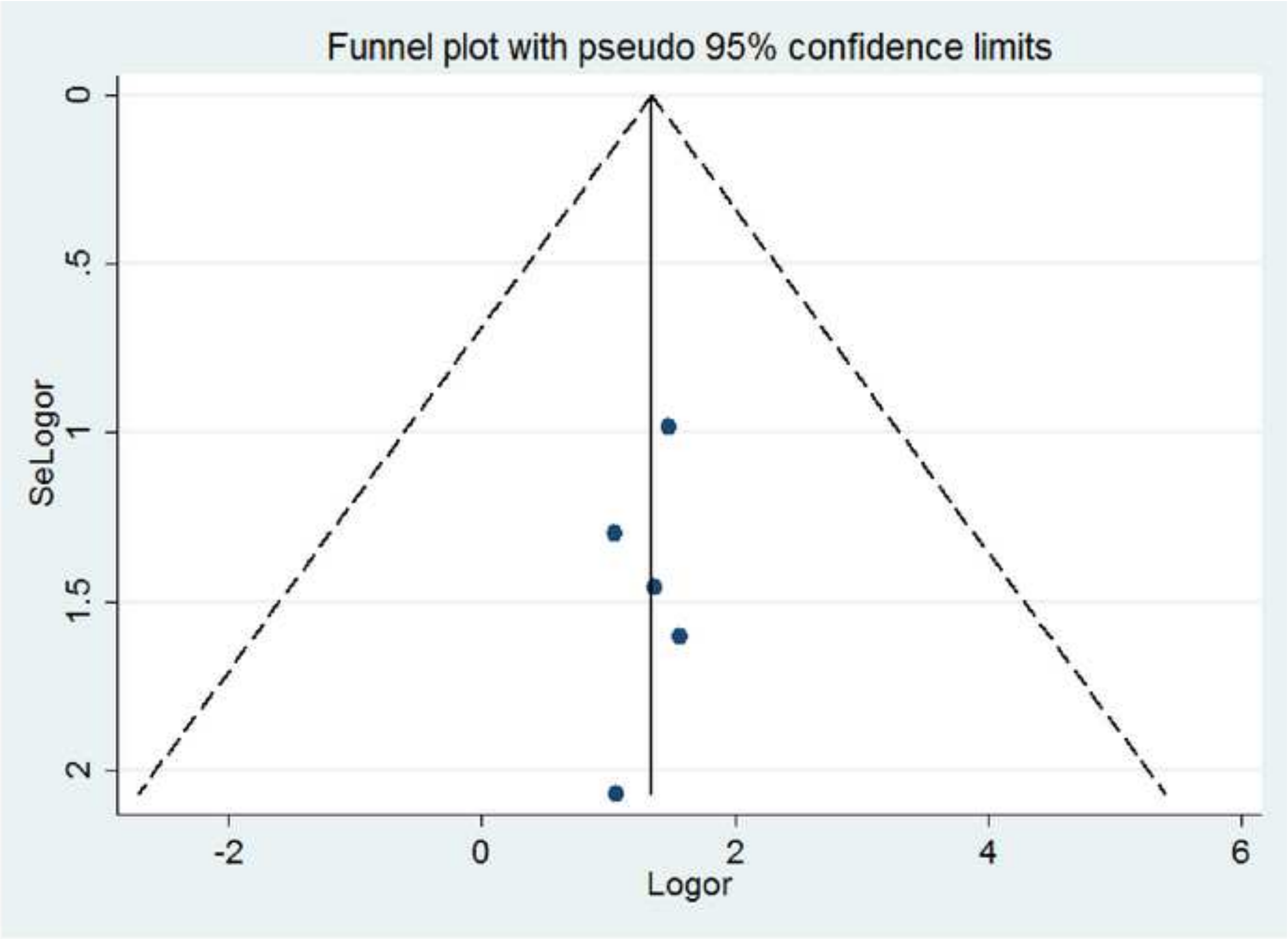

Fig 7: Funnel plot to assess the heterogeneity of the included studies

Click here to access/download
Supporting Information
PRISMA-checklist.docx
