# Supplementary material for: Determinants of diabetic retinopathy in Ethiopia: A systematic review and meta-analysis
Source: PLoS One. 2023 Jun 8;18(6):e0286627. doi: 10.1371/journal.pone.0286627 (PMC10249865; doi:10.1371/journal.pone.0286627)
Supplement: Attachment — Submitted filename: Diabetic retinopathy (SR & MA).docx [file pone.0286627.s003.docx]

**Manuscript Number:** PONE-D-22-31170

**Manuscript Title:** Determinants of Diabetic Retinopathy in Ethiopia; A systematic review and meta-analysis

Congratulations dear authors on your scholarly work; you have brought an important study problem with good findings that have public health importance in the area of practice. However, there are methodological and other concerns that I want you to address before considering the manuscript for publication.

**General comment**

There are multiple typological and grammar usage errors that need extensive proof reading for revisions.

**Specific comments**

## Abstract

1. “Background” should state burden of diabetic retinopathy in Ethiopia. i.e about one fifth of the diabetic patients, 19.48%.
2. Statistics is ok and well described.
3. Conclusions are supported by the findings.

## Introduction:

1. There are redundant ideas and hence the introduction needs further synthesis.
2. What were the unanswered questions, and what will you add to your research?
3. Finally, please include your rationale for doing this research paper.

**Methods**

1. Is the protocol of this review registered? If so, include its registration number. If not, state its current status.
2. I suggest the authors consider using a PICO (possibly with the T or S) format to make the review question explicit and assist with clear specification of the inclusion and exclusion criteria.
3. Provide the primary search string, including the truncation and synonyms as a supplementary file for at least one database.
4. Specify the type of searching strategy (e.g. was it line by line, by combined concepts, did the search include title (TI), abstract (Ab) or full text or all these categories) was used, specify if databases were searched independently and if any modifications were made to the search strategy (e.g. limiters) for different databases. The description of the method should include enough details for reproducibility. Explain the steps in screening (e.g. title, abstract, full text).
5. I suggest reporting the statistics for measurement of the level of agreement for the independent reviews (e.g. quality scores) of each article. Moreover, kindly append a table showing methodological quality of the appraised articles with the last column being ‘overall quality score’.
6. How did you pool the individual knowledge, attitude and practice scores because nearly all the primary studies used to consider their own operational definitions? Please be critical in this issue and support your measurement of these pooled outcomes with sound references.
7. Explain the data extracted from the studies, reliability and validity of data extraction tool. Explain how the variables extracted were determined and criteria for data to be suitable for extraction. Explain if data transformation was required or undertaken when data were reported differently.

**Results**

Please include two columns in Table 1: data collection technique (interview, observation, self administered questionnaire, etc) and funding source for each study (you can say not funded, not reported or name of funder if funded).

**Discussion**

1. The discussion needs to be re-thought as it provides certain explanations that may be inaccurate.
2. There has to be a separate subsection named as “implication of the study” showing beneficence of the findings to the clinicians, decision makers and policy makers.
3. Please include a detailed analysis of the limitations that users should be mindful of interpreting the findings.
