# Supplementary material for: Determinants of diabetic retinopathy in Ethiopia: A systematic review and meta-analysis
Source: PLoS One. 2023 Jun 8;18(6):e0286627. doi: 10.1371/journal.pone.0286627 (PMC10249865; doi:10.1371/journal.pone.0286627)
Supplement: Attachment — Submitted filename: Response to Reviewers.docx [file pone.0286627.s005.docx]

**Authors’ response to editor and reviewer questions and comments**

| **Part I:** | | **Editor Comments** | **Responses** | | | **Remark** | |
| --- | --- | --- | --- | --- | --- | --- | --- |
| 1 | | The grammar needs to be reviewed. The generally acceptable tense for reporting research finding is the past tense. This was not the case in a substantial part of the manuscript. | Ok, thank you. The whole manuscript was edited and corrected using professional language editor | | |  | |
| 2 | | I'm wondering why the author(s) only zero in on three determinants of DR in the manuscript. For a systematic review that seeks to provide evidence for evidence for practice, I think the net should have been cast more widely to consider variables such as age and sex of patient, HBA1C, family history of diabetes, weight/obesity, type of diabetes (T1 or T2), physical activity, occupation etc. | The authors tried to investigate variables that potentially contribute for the occurrence of diabetic retinopathy. But there are few studies reporting on variables such as age and sex of patient, HBA1C, family history of diabetes, weight/obesity, type of diabetes (T1 or T2), physical activity, occupation etc. so in order to avoid small studies bias the authors used those three variables. | | |  | |
| **Part II**: | | **Reviewers comments/questions** |  | | |  | |
| **Reviewer #**1 | | | | | | | |
| **Abstract** | | |  | | | |  |
| 1 | | **Background**” should state burden of diabetic retinopathy in Ethiopia.  i.e about one fifth of the diabetic patients, 19.48%. | it is corrected and stated | | | | line no. 27-28 |
| 2 | | Statistics is ok and well described. | Thank you | | | |  |
| 3 | | Conclusions are supported by the findings. | Thank you | | | |  |
| **Introduction** | | | | | | | |
| 1 | | There are redundant ideas and hence the introduction needs further synthesis. | The whole parts of the introduction are synthesized and restructured | | | whole introduction | |
| 2 | | What were the unanswered questions, and what will you add to your research? | it is incorporated | | | line  93-94 | |
| 3 | | Finally, please include your rationale for doing this research paper. | it is stated | | | Line  94-96 | |
|  | | **Methods** |  | | |  | |
| 1 | | Is the protocol of this review registered? If so, include its registration number. If not, state its current status. | It is registered but it is under reviewed. | | |  | |
| 2 | | I suggest the authors consider using a PICO (possibly with the T or S) format to make the review question explicit and assist with clear specification of the inclusion and exclusion criteria. | The authors used the PICOT/S format, the inclusion and exclusion criteria are well stated. | | | see **table** 1  Inclusion and exclusion criteria | |
| 3 | | Provide the primary search string, including the truncation and synonyms as a supplementary file for at least one database. | **attached at supported files** | | |  | |
| 4 | | Specify the type of searching strategy (e.g. was it line by line, by combined concepts, did the search include title (TI), abstract (Ab) or full text or all these categories) was used, specify if databases were searched independently and if any modifications were made to the search strategy (e.g. limiters) for different databases. The description of the method should include enough details for reproducibility. Explain the steps in screening (e.g. title, abstract, full text). | The searching was made by line by line and combined terms. It was made by title, abstract, full text or all those categories including limiters. Two reviewers independently screened the articles by title, abstract and full text. Any controversy was resolved by discussion.  It is clearly stated in the method section “**Data sources and search strategy” .** | | | Line 104-111 | |
| 5 | | I suggest reporting the statistics for measurement of the level of agreement for the independent reviews (e.g. quality scores) of each article. Moreover, kindly append a table showing methodological quality of the appraised articles with the last column being ‘overall quality score’. | it is included | | | **see table 2** | |
| 6 | | How did you pool the individual knowledge, attitude and practice scores because nearly all the primary studies used to consider their own operational definitions? Please be critical in this issue and support your measurement of these pooled outcomes with sound references. | Since our study focused on factors, outcome ascertainment is not mandatory. Because this study did not focus on “Diabetic retinopathy incidence/prevalence” | | |  | |
| 7 | | 1. Explain the data extracted from the studies, reliability and validity of data extraction tool. Explain how the variables extracted were determined and criteria for data to be suitable for extraction. Explain if data transformation was required or undertaken when data were reported differently. | The format was developed by two reviewers and piloted for its clarity, aim, consistency, and depth of the contents. Simple and consistent codes of response were used. Then the reviewers independently reviewed and extract data from each eligible study. | | | see data extraction section | |
|  |  | 2. **Results:** Please include two columns in Table 1(2): data collection technique (interview, observation, self administered questionnaire, etc) and funding source for each study (you can say not funded, not reported or name of funder if funded). | It is incorporated | | | **Table 2** | |
| **Discussion** | | | | | | | |
| 1 | The discussion needs to be re-thought as it provides certain explanations that may be inaccurate. | | | it is edited and corrected |  | | |
| 2 | There has to be a separate subsection named as “implication of the study” showing beneficence of the findings to the clinicians, decision makers and policy makers. | | | it is stated | Line  269-271 | | |
| 3 | Please include a detailed analysis of the limitations that users should be mindful of interpreting the findings. | | | it is stated | Line  262-264 | | |
