# Supplementary material for: Determinants of diabetic retinopathy in Ethiopia: A systematic review and meta-analysis
Source: PLoS One. 2023 Jun 8;18(6):e0286627. doi: 10.1371/journal.pone.0286627 (PMC10249865; doi:10.1371/journal.pone.0286627)
Supplement: Attachment — Submitted filename: Response to Reviewers.docx [file pone.0286627.s006.docx]

**Response to reviewers for questions and comments**

| **S.no** | **Reviewer comments** | **Response** | **Remark** |
| --- | --- | --- | --- |
| 1 | State the research question using PICO format. | It is stated clearly | Line no: 114-117 |
| 2 | Address the issue whether a protocol for the systematic review was registered and if so report the registration details in the manuscript. | It is registered under the PROSPERO: CRD42023416724 | Line No: 98-100 |
| 3 | In line 246 of the revised manuscript, please replace "don't" with "do not". | It is edited and corrected. | Line No. 243-246 |
| 4 | Rework the implication of the study to indicate clearly what the implications of the results are for clinicians, patients and decision makers. | it is clearly stated | Line No: 267-273 |
